# Biomimetic cardiac tissue chip and murine arteriovenous fistula models for recapitulating clinically relevant cardiac remodeling under volume overload conditions

**DOI:** 10.3389/fbioe.2023.1101622

**Published:** 2023-02-16

**Authors:** Tatyana Isayeva Waldrop, Caleb Graham, William Gard, Kevin Ingle, Travis Ptacek, Nguyen Nguyen, Bailey Lose, Palaniappan Sethu, Timmy Lee

**Affiliations:** ^1^ Department of Medicine and Division of Nephrology, University of Alabama at Birmingham, Birmingham, AL, United States; ^2^ Division of Cardiovascular Disease, University of Alabama at Birmingham, Birmingham, AL, United States; ^3^ Department of Biomedical Engineering, University of Alabama at Birmingham, Birmingham, AL, United States; ^4^ Center for Clinical and Translational Science, University of Alabama at Birmingham, Birmingham, AL, United States; ^5^ Veterans Affairs Medical Center, Birmingham, AL, United States

**Keywords:** tissue chip, cardiac remodeling, tissue engineering, arteriovenous fistula, heart failure, end stage renal disease

## Abstract

Cardiovascular events are the primary cause of death among dialysis patients. While arteriovenous fistulas (AVFs) are the access of choice for hemodialysis patients, AVF creation can lead to a volume overload (VO) state in the heart. We developed a three-dimensional (3D) cardiac tissue chip (CTC) with tunable pressure and stretch to model the acute hemodynamic changes associated with AVF creation to complement our murine AVF model of VO. In this study, we aimed to replicate the hemodynamics of murine AVF models *in vitro* and hypothesized that if 3D cardiac tissue constructs were subjected to “volume overload” conditions, they would display fibrosis and key gene expression changes seen in AVF mice. Mice underwent either an AVF or sham procedure and were sacrificed at 28 days. Cardiac tissue constructs composed of h9c2 rat cardiac myoblasts and normal adult human dermal fibroblasts in hydrogel were seeded into devices and exposed to 100 mg/10 mmHg pressure (0.4 s/0.6 s) at 1 Hz for 96 h. Controls were exposed to “normal” stretch and experimental group exposed to “volume overload”. RT-PCR and histology were performed on the tissue constructs and mice left ventricles (LVs), and transcriptomics of mice LVs were also performed. Our tissue constructs and mice LV both demonstrated cardiac fibrosis as compared to control tissue constructs and sham-operated mice, respectively. Gene expression studies in our tissue constructs and mice LV demonstrated increased expression of genes associated with extracellular matrix production, oxidative stress, inflammation, and fibrosis in the VO conditions vs. control conditions. Our transcriptomics studies demonstrated activated upstream regulators related to fibrosis, inflammation, and oxidative stress such as collagen type 1 complex, TGFB1, CCR2, and VEGFA and inactivated regulators related to mitochondrial biogenesis in LV from mice AVF. In summary, our CTC model yields similar fibrosis-related histology and gene expression profiles as our murine AVF model. Thus, the CTC could potentially play a critical role in understanding cardiac pathobiology of VO states similar to what is present after AVF creation and may prove useful in evaluating therapies.

## Introduction

There are over 800,000 patients in the United States with End Stage Kidney Disease (ESKD), and approximately 70% of these patients utilize hemodialysis as their primary form of kidney replacement therapy (United States Renal Data System, 2021). Thrice-weekly hemodialysis remains the most commonly selected kidney replacement modality (United States Renal Data System, 2021). The arteriovenous fistula (AVF) is the preferred form of vascular access for hemodialysis, as central venous catheters and AV grafts are more prone to infection and loss of patency, leading to increased costs and mortality (Lee, 2017). However, AVF creation (i.e., surgically connecting a high blood flow artery with a low pressure, high capacitance vein) results in an acute decrease in systemic vascular resistance due to blood bypassing arterioles, followed by an increase in venous return and sympathetic signaling, leading to increased cardiac output (CO) (Basile et al., 2008; Reddy et al., 2016; Malik et al., 2021). The increased ventricular filling and CO following AVF creation produces structural and functional cardiac changes, such as left ventricular (LV) remodeling and development of LV hypertrophy (Dundon et al., 2014; Alkhouli et al., 2015; Mathew et al., 2019; Paré et al., 2022).

Our recent work assessed cardiac structure and function in our murine AVF model where we observed a significant increase in CO and LV end diastolic diameter (LVEDD) at 7 and 21 days in AVF mice, as compared to sham-operated mice, and a significant decrease in ejection fraction (EF) and fractional shortening (FS) at 21 days in the AVF group (Ingle et al., 2021). Furthermore, we also showed development of LV fibrosis 21 days after AVF creation. Thus, our murine AVF model recapitulates the volume-overload state that is seen after clinical AVF creation.

Although animal models have several unique advantages for studying cardiac remodeling, they also have limitations. For example, volume- and/or pressure-overload observed in various cardiovascular disease states are associated with different hemodynamic loading profiles impacting the myocardium. Although *in vitro* models cannot offer the holistic picture provided by animal models, they can offer greater control over hemodynamic parameters as well as ease of access to the biological material of interest for downstream assays. Thus, we have adapted a biomimetic cardiac tissue chip (CTC) model to recapitulate the pre- and post-AVF murine hemodynamics in order to serve as a complementary tool to our animal AVF model. Using 3D engineered tissue containing cardiac myoblasts and fibroblasts, we have previously demonstrated substantial changes in the cell morphology and gene profiles after 48 h of hemodynamic stresses associated with pressure or volume overload (Rogers et al., 2019).

In our present study, we investigated mechanisms of cardiac remodeling related to volume overload using a murine AVF model at 28 days after AVF creation and showed that critical hallmarks of pathophysiological remodeling, including fibrotic remodeling and associated changes in gene expression, can be replicated using both *in vitro* CTC studies and an *in vivo* mouse model.

## Materials and methods

### Cell culture

H9c2 myoblasts (ATCC (H9c2(2-1); ATCC, CRL-1446), derived from embryonic rat cardiac tissue (Brandt et al., 1976; Kimes and Brandt, 1976) were cultured in Dulbecco’s modified Eagle’s medium (DMEM; ATCC, 30-2002) supplemented with 10% fetal bovine serum (FBS; Fisher, FB12999102) and 1% penicillin/streptomycin (P/S; Life Technologies, 15140- 122) in 5% CO2 at 37°C in a humidified incubator. Primary human dermal fibroblasts (ATCC, PCS-201-010) were cultured under the same specifications.

### Cardiac tissue chip Fabrication

The CTCs utilized in this experiment were fabricated similarly to previous iterations (Budhathoki et al., 2021) using polydimethylsiloxane (PDMS; QSil 216, Quantum Silicones, Richmond, VA, United States) and soft lithography. Square frames (5 cm × 5 cm × 1 cm in height) with a central circular cavity (2.8 cm in diameter) were generated using replica molding and PDMS mixed at a 10:1 base-to-cross-linker ratio followed by heat-curing. Flexible membranes were made by spin-coating PDMS mixed at a 15:1 base-to-crosslinker ratio onto silicon wafers and then heat-curing. Next, six PDMS posts (1 cm in height × 0.1 cm in diameter) were attached to each membrane using 3D-printed stencils for precise positioning. The posts were arranged into three pairs, measuring 8 mm from center-to-center with a 6 mm space between adjacent pairs.

Following cleaning, the frames and post-laden membranes were bonded with oxygen plasma (Harrick Plasma Systems, Ithaca, NY, United States) using 700 mTorr pressure, 30 W, and 30 s exposure time. The freshly bonded devices were heated further to strengthen the bond and eliminate any remaining air pockets between the frame and membrane. Lastly, the devices were carefully peeled from the silicon wafers and excess membranes were trimmed.

### Cardiac tissue chip seeding

**FIGURE 1:**
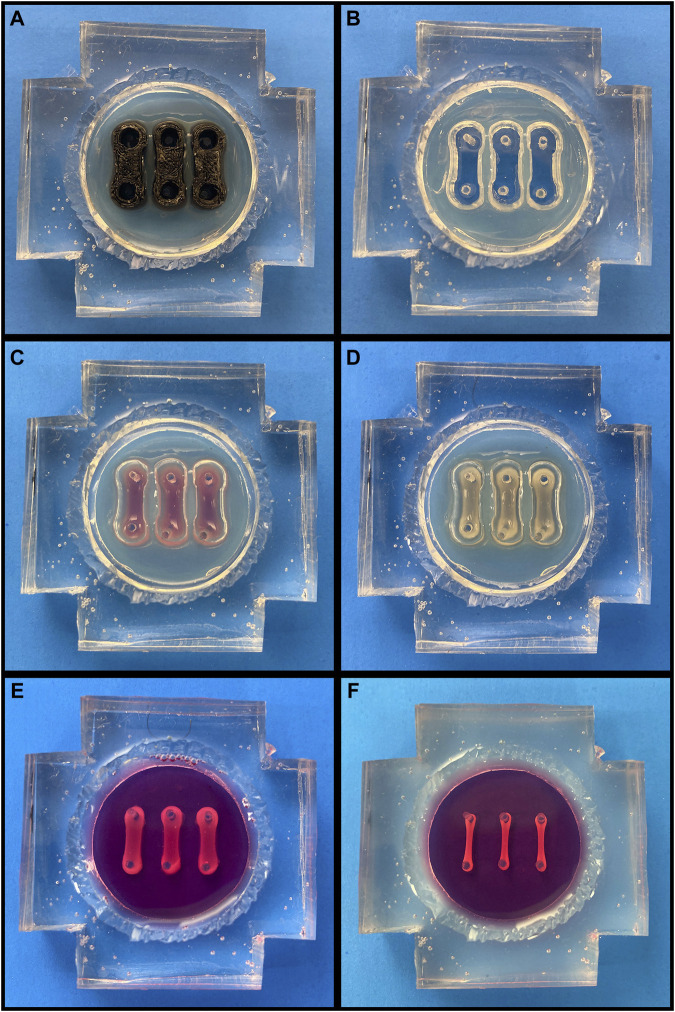
Cardiac Tissue Chip (CTC) Preparation and Seeding. (A-F, going left to right, top to bottom). **(A)** Agarose-filled CTC with trough molds in place. **(B)** CTC following agarose solidification and mold removal. **(C)** CTC with freshly seeded cell/hydrogel suspension. **(D)** CTC following gelation. **(E)** CTC following agarose removal and media filling. **(F)** CTC after engineered cardiac tissues have compacted over 72 h.

3D-printed dumbbell-shaped molds were placed in each device (one per pair of posts) and then the device chambers were filled with 3.5% molten agarose (Figure 1A). After agarose gelling at room temperature, the molds were removed, leaving behind three empty troughs (Figure 1B). Following a PBS wash, the devices were filled with a 1% w/v solution of bovine serum albumin (BSA; Sigma-Aldrich, A7906) in PBS and stored overnight at 4°C to generate a non-adherent coating. Prior to seeding, the devices were warmed to 37°C and the BSA solution was removed.

With the devices now prepared, the H9c2s were dissociated with 0.25% Trypsin/EDTA (Gibco, 25200-056) and resuspended with the human dermal fibroblasts, which were dissociated with 0.05% Trypsin/EDTA (Gibco, 25300-054). Each 200 μL cardiac tissue construct contained 2 × 106 H9c2s and 0.667 × 106 fibroblasts and was generated as follows: first, thrombin (Millipore, 605195) was added to 40 mM CaCl2, and 10 μL of the resulting 0.048 units/μL thrombin solution was pipetted into a microcentrifuge tube (one tube per tissue construct). After the cells had been combined in the appropriate ratio and pelleted, they were resuspended in a 10% v/v solution of growth factor reduced Matrigel (Corning, 354230) in DMEM supplemented with 10% FBS and 1% P/S. 180 μL of this cell/hydrogel suspension was then added to each thrombin-containing tube. Lastly, proceeding one tube at a time to prevent premature gelling, 10 μL of 40 mg/mL fibrinogen (Millipore, 341576) was added to each tube, the suspension was gently pipetted to homogenize the mixture, and the 200 μL suspension was pipetted into one of the empty troughs (Figure 1C). Each tissue construct had a final concentration of 2 mg/mL fibrin, 1.2 units thrombin/mg fibrin, and 2 mM CaCl2. After seeding, the CTCs were placed into the 37°C humidified incubator at 5% CO2 for 60-75 min, allowing the tissue constructs to gel (Figure 1D). Following this, the agarose was removed, the device chamber was washed with PBS to remove any agarose debris, and DMEM containing 10% FBS, 1% P/S, and 2 mg/mL aminocaproic acid (ACA; Acros, 103301000) was added to each (Figure 1E). The tissue constructs were maintained in static culture for 72 h with daily media changes prior to experimentation to allow for compaction and ECM deposition (Figure 1F).

### Cardiac tissue chip volume overload experiments

**FIGURE 2:**
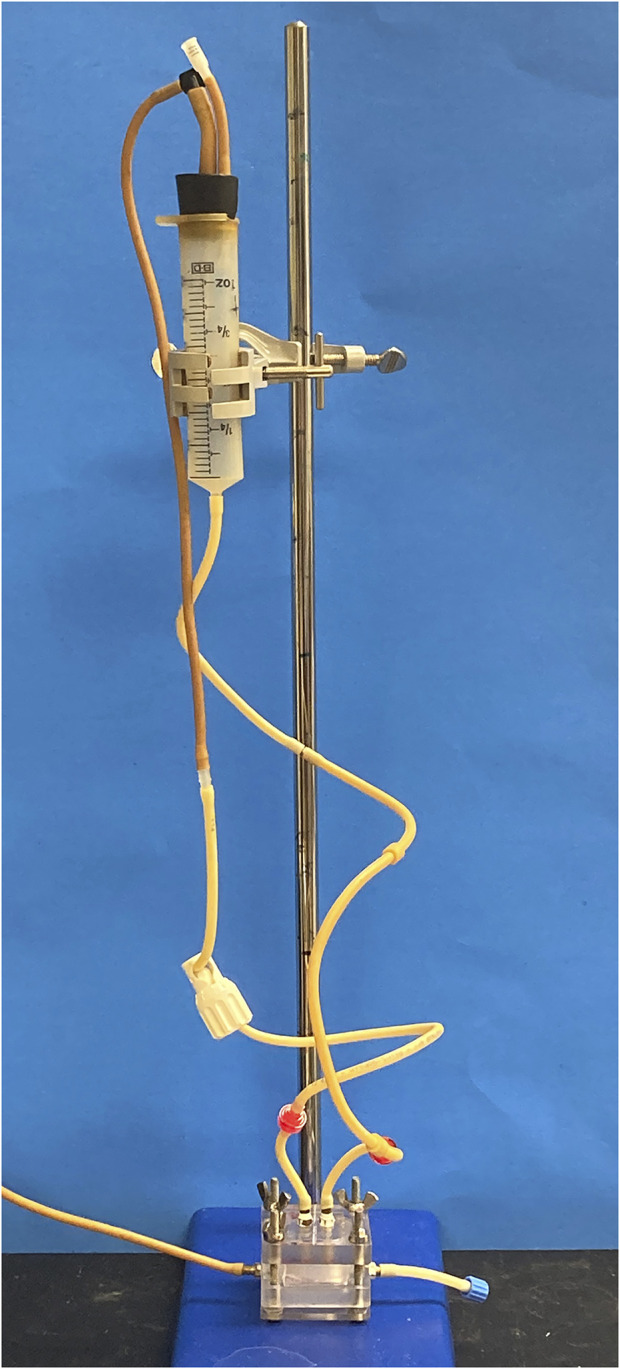
CTC Dynamic Flow Loop. Media fills the modified 50 mL syringe and leaves through the bottom, where it is attached to the CTC media inlet port. End-diastolic pressure is determined by the height separating the top of the media column and the media chamber. One-way valves (red) on the media tubing prevent retrograde flow. Media outflow resistance can be tuned *via* the white resistance element on the outflow tubing, which affects the end-systolic position of the CTC membrane and length of the engineered cardiac tissues. The tubing in the bottom left connects the pneumatic pump to the bottom polycarbonate piece air chamber.

A modified version of the Biomimetic Cardiac Tissue Model (BCTM) designed by our laboratory (Rogers et al., 2019; Budhathoki et al., 2021) was used in these experiments to mimic left intraventricular pressure and volume changes. First, the seeded CTC was sandwiched between an upper polycarbonate piece (5 cm × 5 cm × 1 cm in height) with media inlet and outlet ports, and a bottom polycarbonate piece of equal size that had a 3 cm × 3 cm × 0.5 cm deep centrally-located cavity with inlet and outlet ports for pressurized air. A 2 cm × 0.9 cm × 0.4 cm hemicylindrical PDMS fragment was also placed in the cavity of the bottom piece. Next, flexible tubing with one-way valves to ensure anterograde flow was used to connect the sandwiched CTC to a media reservoir, and a peristaltic pump was employed to prime the device/reservoir loop. Following priming, the CTC loops were moved to a 37°C humidified incubator filled with 5% CO2, and the media reservoirs were clamped to ring stands and positioned at appropriate heights. The end-diastolic pressure in this system (Figure 2), is dependent upon the hydrostatic pressure generated by the height of the media column as well as the compliance of the PDMS membrane. In order to facilitate greater stretch in the volume overload (VO) devices in comparison to the normal load (NL) devices, the VO device membranes were fabricated with 200 μL of PDMS, whereas the NL devices were fabricated with 500 μL. End-diastolic pressures of 10 mmHg were established by raising the media column to a height of 32 cm for the NL devices and 55 cm for the VO devices. Next, the bottom polycarbonate piece of each CTC was connected to a pneumatic pump (LB Engineering GbR, Berlin, Germany) set at 60 bpm and pulse duration of 0.4 s, which generated peak systolic pressures of ∼100 mmHg. This systolic pulse below the membrane returns it to a position closer to the non-deformed baseline, propelling the media forward, and the ensuing 0.6 s filling phase pushes the membrane downward, stretching it and the tissue constructs around the hemi-cylindrical obstacle below. While these LV pressures are consistent with those seen in mice (Joho et al., 2007), a sub-physiological heart rate of 60 bpm was chosen due to technical limitations of the employed pneumatic pumps and to allow for full stretch and relaxation of the tissue constructs. Lastly, a tunable resistance element on the media outflow tubing allowed for control over the degree to which the PDMS membrane returned to its non-deformed baseline at the end of systole. For the NL devices, these elements were tuned such that the membrane was flat at the end of systole, while those of the VO CTCs were tuned to maintain residual strain (in comparison to the device resting length) at the end of systole. Based on imaging measurements, the NL tissue constructs experienced ∼9% strain, calculated as [(end-diastolic length–end-systolic length)/end-diastolic length whereas the VO constructs experienced ∼18%. The tissue constructs were subjected to these hemodynamic parameters for 96 h before being harvested for molecular studies or histology.

### Creation of murine arteriovenous fistula

**FIGURE 3:**
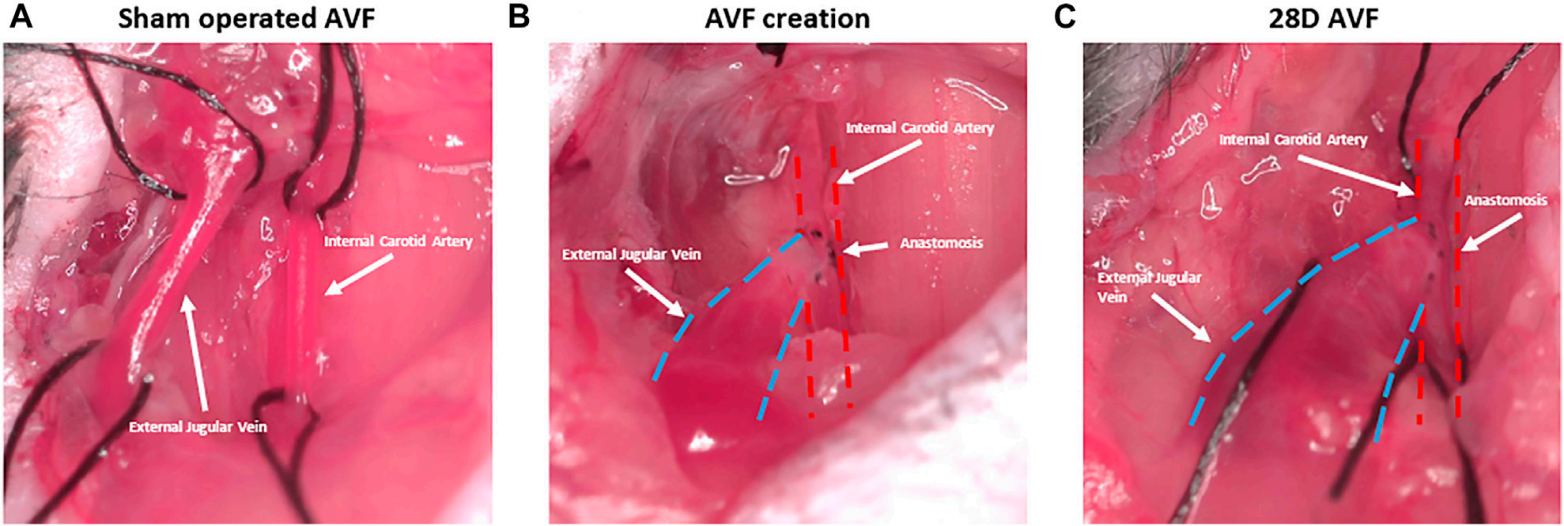
Representative figure depicting stages of arteriovenous fistula (AVF) creation. **(A)** Sham operated AVF lacking anastomosis between external jugular vein and internal carotid artery. **(B)** AVF creation with anastomosis between artery and vein. **(C)** AVF at 28D post-surgery and dilation of the AVF vein is observed.

All animal procedures and experiments were approved by the University of Alabama at Birmingham Institutional Animal Care and Use Committee and conformed with the principles of the National Institutes of Health Guide for the Care and Use of Laboratory Animals. In our study we used eight C57BL/6 male mice (Taconic Biosciences, Hudson, NY). The AVFs were created by connection of the jugular vein to the side of carotid artery, as previously published (Pike et al., 2017; Ingle et al., 2021; Somarathna et al., 2021) (Figure 3). Mice were 10–11 weeks old at the time of AVF creation. Age-matched sham-operated mice served as the control group. For this study, we measured end timepoint cardiac parameters. All mice had baseline 2D echocardiography performed at 10 weeks of age, and at the time of sacrifice at 28 days post-surgery. Hearts were harvested at 28 days after AVF creation in experimental group and corresponding control group. The LVs were dissected and preserved for histology and RNA analysis.

### Echocardiography and image analysis

Cardiac function in the C57BL/6 mice were evaluated using a VEVO 2100 micro-ultrasound system with a 24–30 MHz bi-frequency transducer (Visual Sonics, Toronto, Canada) as previously described in detail and published (Ingle et al., 2021). To characterize the LV systolic function we calculated EF and FS, with details as previously published (Ingle et al., 2021). We measured CO to measure the rate of blood flowing from the heart with details as previously published (Ingle et al., 2021).

### Trichrome and picrosirius red staining

Several morphometry techniques were employed to stain collagen in the LV, including Picrosirius red staining (PSR) under bright light and polarized light, and combination of Verhoeff’s elastic and Masson’s trichrome staining. The mouse heart tissues and cardiac tissue constructs were fixed in 10% formalin and processed for paraffin embedding. Sections of 5 µm (Leica RM 2135) were applied to charged slides, deparaffinized, and hydrated with distilled water. Slides were stained as previously described (O’Connor and Valle, 1982). The elastic fibers are stained black by Verhoeff’s technique, the muscle and connective tissue stained red and green/blue, respectively, by a modification of Masson’s trichrome, and cell nuclei stain blue-black with iron hematoxylin.

Cardiac tissue constructs and LV tissues from 28D sham and AVF mice were collected to be analyzed for fibrosis. The presence of collagen in the LV sections was evaluated in the interstitial area and perivascular areas. Images were captured using an Olympus IX73 Brightfield microscope. For each sample, 4-10 images were taken, and the collagen stain was analyzed by imaging software (CellSens dimensions (Olympus version 3.1) as mentioned in our previous work (Ingle et al., 2021). In brief, interstitial fibrosis and collagen deposition in the LV sections and cardiac tissue constructs, was quantified by first selecting a threshold range. All images were quantified using this same threshold. The positive staining was then calculated as a percentage of the total image area. For quantifying perivascular fibrosis, a region of interest was traced around the vessel such that the lumen and surrounding tissue were not included in the calculation. The stained area within the region of interest was calculated as a percentage of the region of interest.

### Real-time polymerase chain reaction (RT-PCR)

Total RNA was isolated using the miRNA isolation kit (Qiagen, cat #74106) according to the protocol of the manufacturers. Samples were treated with DNAse I (Qiagen cat# 79254). RNA was quantitated using Epoch/Take3 spectrophotometer system (BioTek Instruments, Inc.). Complementary DNA was generated with iScript cDNA Synthesis kit (Qiagen). We used StepOnePlus™ Real-Time PCR System to run a Taqman gene expression assay. Taqman expression assay probes for human genes have cross reactivity with corresponding rat genes expressed by H9c2 cells. To prepare the PCR reaction mix for each Taqman gene expression assay and samples on cycling plate: for one sample combine 10 µl of 2x Taqman fast universal PCR master mix, 1 µL Taqman gene expression assay, 5 µL water with 4 µL cDNA mix.

### Transcriptomic studies and bioinformatic analysis

LV was harvested from AVF mice and sham-operated mice at postoperative day 28 and frozen with liquid nitrogen and stored at -80 °C. Isolation of total RNA and RNA sequencing were performed by Discovery Life Sciences (Huntsville, AL). Isolation and preparation of RNA was performed by Discovery Life Sciences standard protocols, and details of quality control assessment and library preparation were previously published (Somarathna et al., 2021). RNA libraries were sequenced on a NovaSeq 6,000 instrument (Illumina, San Diego, CA) at a 150 base pair read length in order to achieve 50 M paired-end reads (100 M total reads).

Reads were quality controlled by FASTQC 0.11.5 (Andrews, 2015). Adaptors were trimmed from reads using cutadapt 1.13.1 (Martin, 2011). Adaptor trimmed reads were aligned to the mm10 reference genome using STAR 2.5.3 (Dobin et al., 2012). Transcript quantification and calculation of differential gene expression were performed using DESeq2 1.18.1 in R 4.1.2 (Love et al., 2014). Ingenuity pathway analysis (IPA) (Krämer et al., 2014) was used to detect canonical pathways associated with differentially expressed genes, the activation or inhibition of upstream regulators and novel pathways and networks of differentially expressed genes.

### Statistical analysis

All data was expressed as means and standard deviation of means. Mann-Whitney U test analysis was used to compare the control and AVF groups for the echocardiographic measurements. Multiple t-test analysis was used to compare the VO and NL conditions for RT-PCR studies. Results for perivascular and interstitial PSR staining were analyzed using unpaired t-test. GraphPad Prism version 9 (La Jolla, CA) was used for statistical analysis and *p* < 0.05 was considered statistically significant. Statistical tests for differential expression were performed using the built-in method in DESeq2. Genes with a FDR corrected *p* < 0.05 were considered significant.

## Results

Our studies incorporate both *in vitro* and *in vivo* models. We have previously characterized our mouse AVF model at 7 and 21 days (Ingle et al., 2021) and in this study we present new results that characterize our AVF model at 28 days after AVF creation from RT-PCR, transcriptomics, and histology studies. In parallel, we characterized tissue constructs from the CTC to develop a model that can recapitulate findings seen in murine AVF model at 28 days.

### *In vivo* murine AVF model studies

#### Heart weights

We measured left ventricle (LV) and right ventricle (RV) weight in both groups of mice normalized by tibia length, which remains constant after maturity (Yin et al., 1982). At the day of sacrifice in the AVF group, the ratio of LV mass to the tibial length and RV to the tibial length were significantly higher compared to sham-operated control mice (*p* < 0.001 and *p* < 0.05, respectively) (Table 1).

**TABLE 1:** Measurements of tibia length, left and right ventricle masses, and the ratio between the ventricle masses and tibia lengths. Values represent mean ± SD.

​	Sham operated D28​	AVF D28​	​Probability Value
Tibial Length (mm)​	17.17​±1.213	17.74​​±0.2992	​ns
LV (mg)​	85.5​±6.35​	104.5​​±4.110	​*p* < 0.05​
RV (mg)​	19.7​​±3.78	27.1​±3.46​	​​*p* < 0.05​
LV/TL (mg/mm)​	5.0 ± 0.25​	5.9 ± 0.13​	*p* < 0.01​
RV/TL (mg/mm)​	1.1±​0.14	1.5 ± 0.18​	*p* < 0.05​

#### Echocardiographic measurements

**FIGURE 4:**
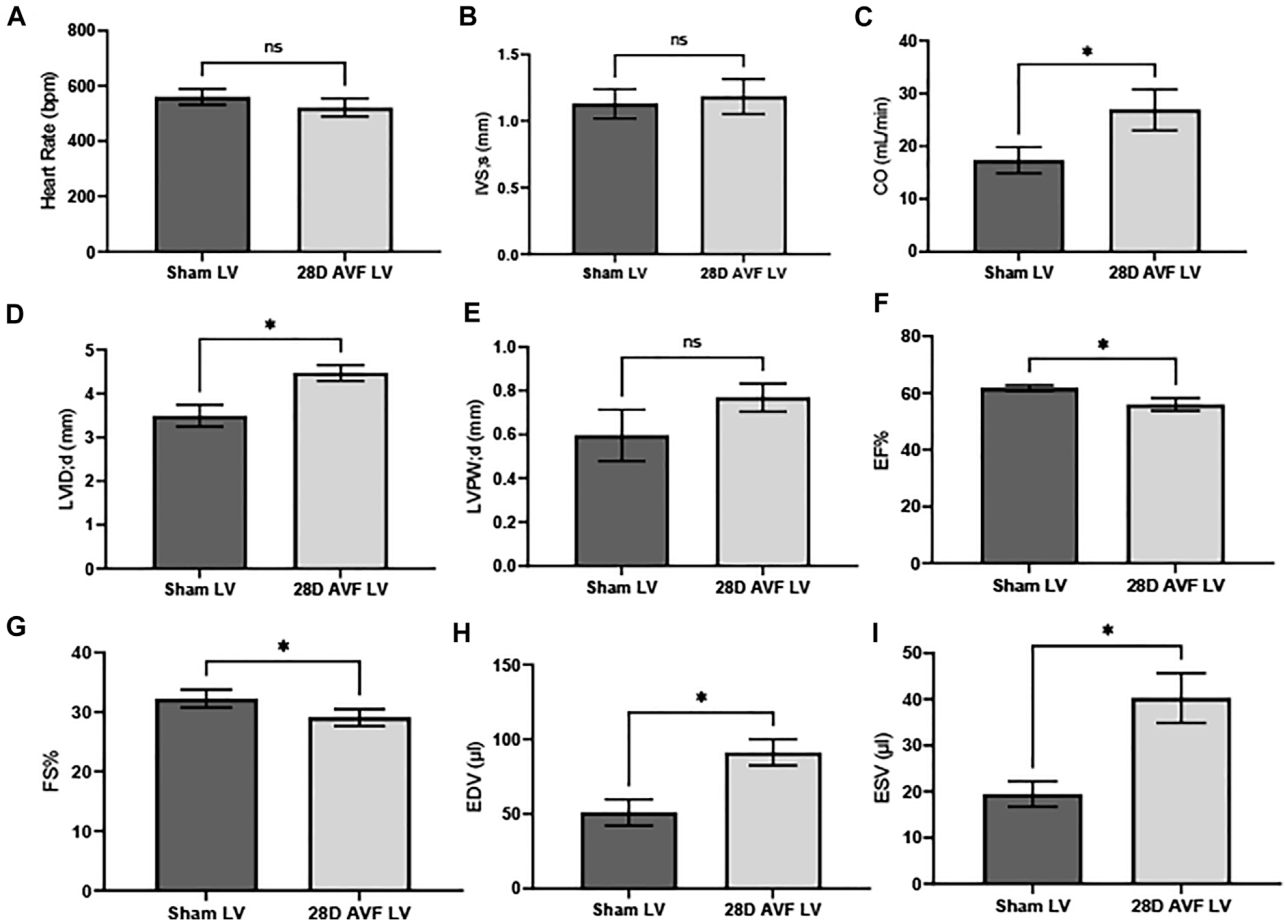
Cardiac Parameters for sham and AVF Mice 28D after surgery: **(A)** heart rate (HR), **(B)** interventricular septal end diastole (IVS;d), **(C)** cardiac output (CO), **(D)** left ventricular internal diameter end diastole (LVID;d), **(E)** left ventricular posterior wall end diastole (LVPW;d), **(F)** ejection fraction (EF), **(G)** fractional shortening (FS), **(H)** end diastolic volume (EDV), **(I)** end systolic volume (ESV). Data was analyzed using a Mann-Whitney U test GraphPad Prism version 8.0 (La Jolla, CA). ns - no-significance; (*) *p* ≤ 0.05. N = 4 for each group. Values represent mean ± S.D.

Baseline echocardiography was performed before the AVF or sham-operated surgery. No significant differences in the function and structure of the heart were observed (Supplementary Figure S1). Echocardiograms performed on the contralateral vessels and AVF indicated that the AVF was patent at 28 days post surgery (Supplementary Figure S2). However, at 28 days multiple cardiac parameters from the AVF group were impaired, as compared to the sham operated group (Figure 4). We observed significant differences in heart dimensions between the AVF- and sham-operated groups. Both LV end-systolic (LVID;s) and end-diastolic inner dimension (LVID;d) in the AVF-operated mice were significantly higher compared to the sham-operated group (*p* < 0.05). Furthermore, end diastolic and end systolic volume were significantly greater in the AVF group (*p* < 0.05). To characterize cardiac function, we calculated EF, FS, and CO. LV from the AVF group exhibited a progressive decline of FS and EF, while also displaying a significant increase in LV chamber dilation and CO (*p* < 0.05). Representative raw echocardiogram data for both the left ventricle, contralateral blood vessels, and AVF is shown in Supplementary Figures S3–S11.

#### Evaluation of collagen deposition

**FIGURE 5:**
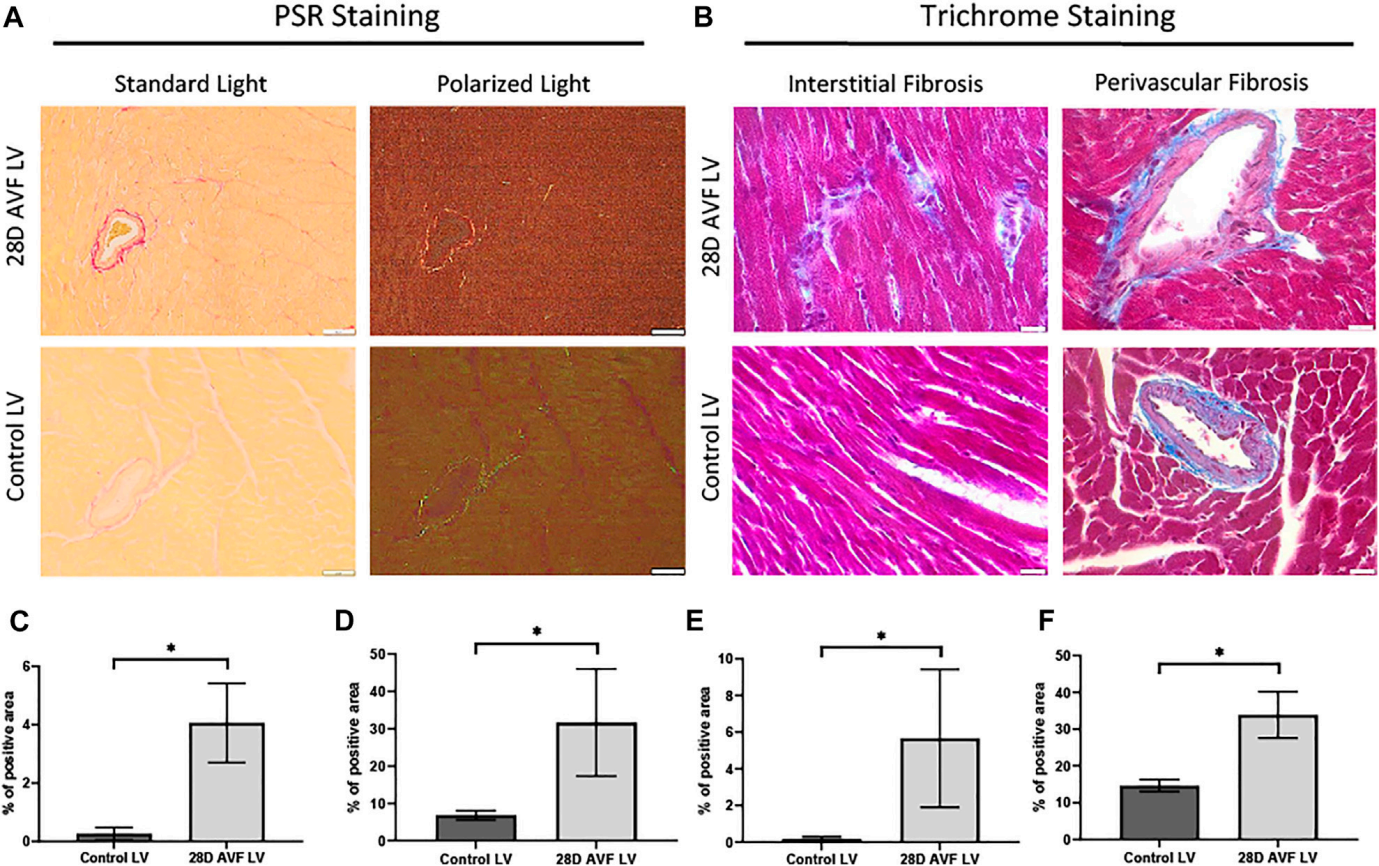
Histological staining performed on control left ventricle and 28D AVF left ventricle. **(A)** Picrosirius red staining (PSR) performed on control LV 28D AVF tissue under standard light and plane polarized light (×20 magnification, scale bar 50 µm). **(B)** Masson’s trichrome (MT) staining performed on control LV and AVF tissue (×40 magnification, scale bar 20 µm). Specific sections of interstitial and perivascular fibrosis are also shown. **(C)** Graph showing percentage of interstitial area stained by PSR. **(D)** Graph showing percentage of perivascular area stained by PSR. **(E)** Graph showing percentage of interstitial area stained by MT. **(F)** Graph showing percentage of perivascular area stained by MT (*) *p* ≤ 0.0001. N = 4 for each group. Values represent mean ± S.D.

To detect and quantify collagen deposition, we analyzed PSR staining images from cardiac histological sections using both bright field and polarized light. Both interstitial and perivascular collagen deposition were shown to be greater in the LV of the AVF mice as compared to sham operated mice (4.0% vs. 0.3% and 31.6% vs. 6.8%, respectively; p < 0.0001; Figure 5). Under polarized light, the collagen fibrils in the LV of the sham-operated group ranged in color from green to light yellow. Collagen fibrils observed in the AVF group sections displayed intense yellow-red staining which is indicative of greater collagen density (Figure 5).

#### Gene expression studies with RT-PCR

**FIGURE 6:**
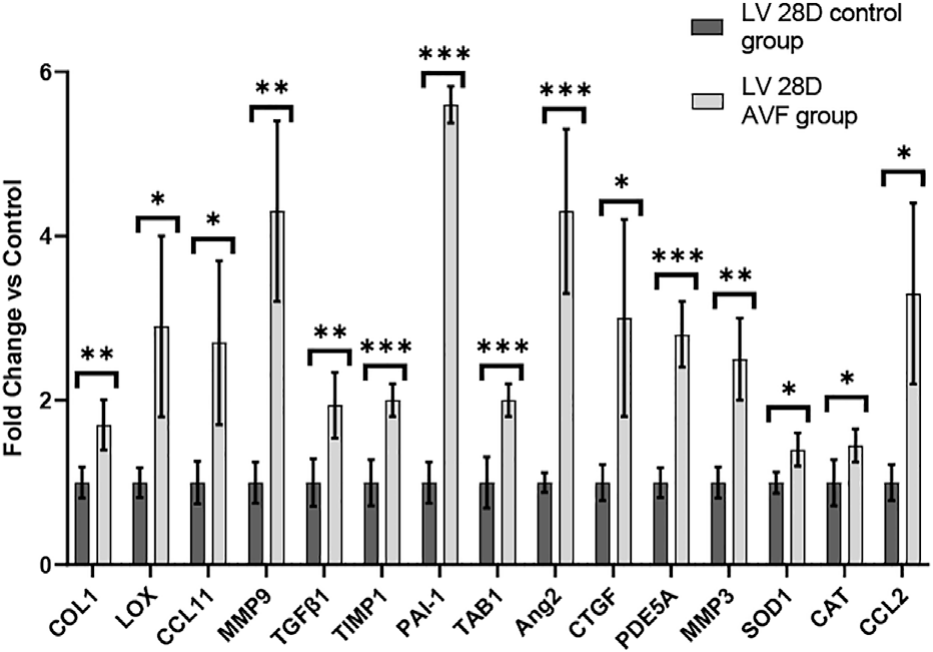
Gene Expression Profiles in 28-day Murine AVF Model vs. Sham Operated AVF. *p < 0.05, **p < 0.01, and ***p < 0.001. N = 4 for each group. Values represent mean ± S.D.

To study gene expression patterns in response to AVF creation, RT-PCR was performed. RT-PCR was performed on LV tissue of 28 days hearts of mice with AVF creation and sham-operated control (Figure 6). Our results demonstrated that genes associated with fibrosis, inflammation, and oxidative stress were significantly differentially expressed in our AVF mice, as compared to sham-operated control, at 28 days. The list of primers used can be found in Supplementary Table S1.

#### Transcriptomics studies of 28 days AVF vs. sham-operated AVF left ventricle

**FIGURE 7:**
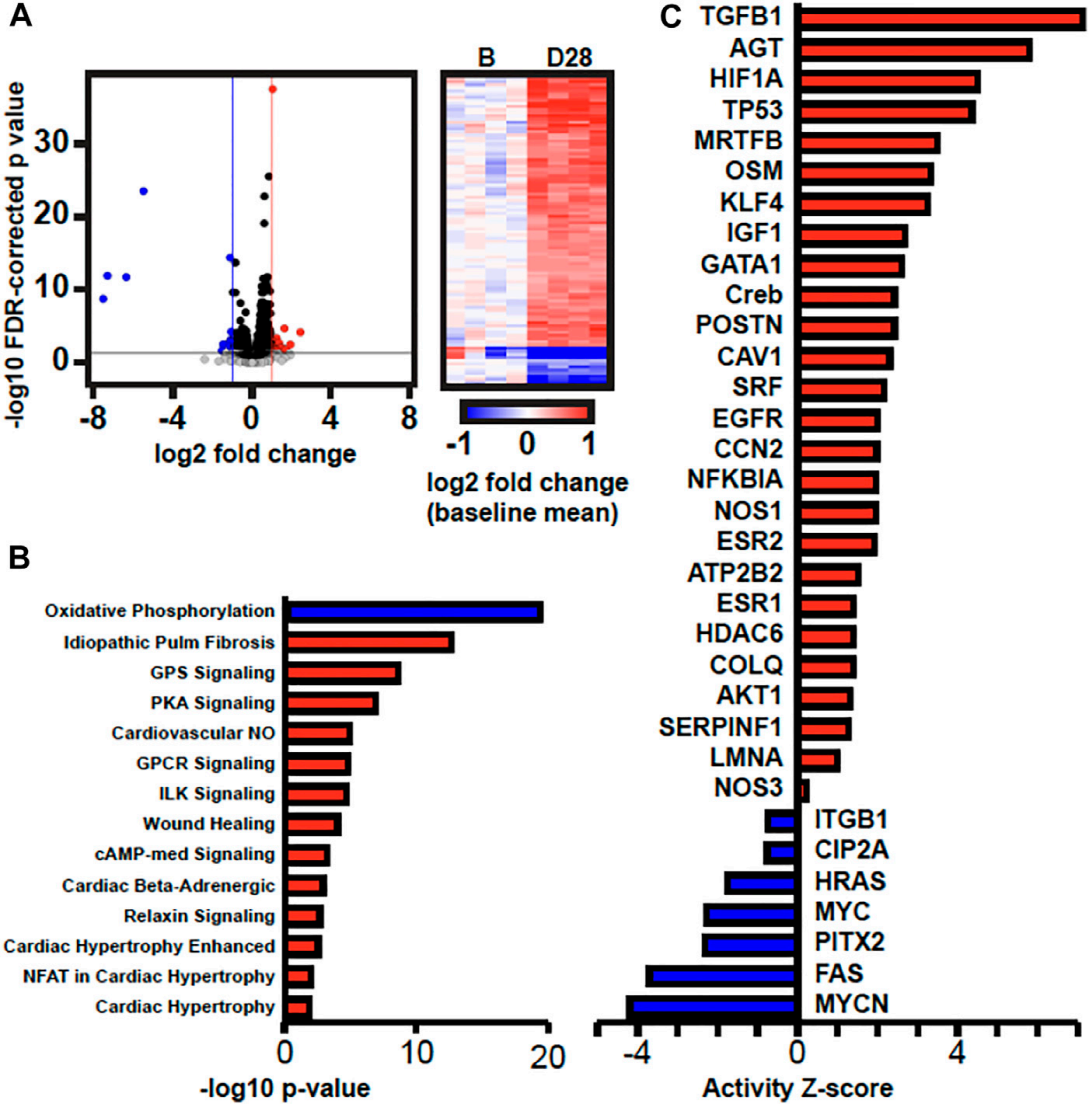
Transcriptomics Analysis Comparing Sham-operated vs. 28 Da y AVF. **(A)** Volcano plot of gene expression results and heatmap of DEGs. In the volcano plot, the horizontal black line marks *p* = 0.05, the vertical blue and red lines mark log2FC = -1 and 1, respectively. In the heatmap, gene expression in each sample is represented as log2 fold change relative to the mean expression of the four baseline samples. **(B)** Canonical pathways and **(C)** upstream regulators that are predicted to have increased or decreased activity (red and blue, respectively) by IPA.

We next performed transcriptomics analysis using RNA-seq to identify potential mechanisms and pathways to explain the observed changes in gene expression and histology in 28 days AVF LV as compared to sham-operated LV. We sequenced an average 82 million read pairs per sample (73–91 million read pairs), of which 58% (range 54%-63%) were uniquely aligned in proper pairs to the mouse genome. We subsequently identified 938 genes significantly differentially expressed between AVF and sham-operated group using a 5% FDR (q < 0.05). 651 were upregulated and 287 downregulated in AVF group (Figure 7A). Ingenuity Pathway Analysis (IPA) of differentially expressed genes predicted activation of pathways associated cardiac hypertrophy signaling, cardiac beta adrenergic signaling, and pulmonary fibrosis idiopathic signaling and deactivation of the oxidative phosphorylation pathway (Figure 7B). IPA was also able to predict activation and inhibition of upstream regulators. Placement of AVF was significantly predicted to activate many upstream regulators related to fibrosis, extracellular matrix production, and cell proliferation such as TGFβ1, CCN2, IGF1, and EGFR (Figure 7C).

**FIGURE 8:**
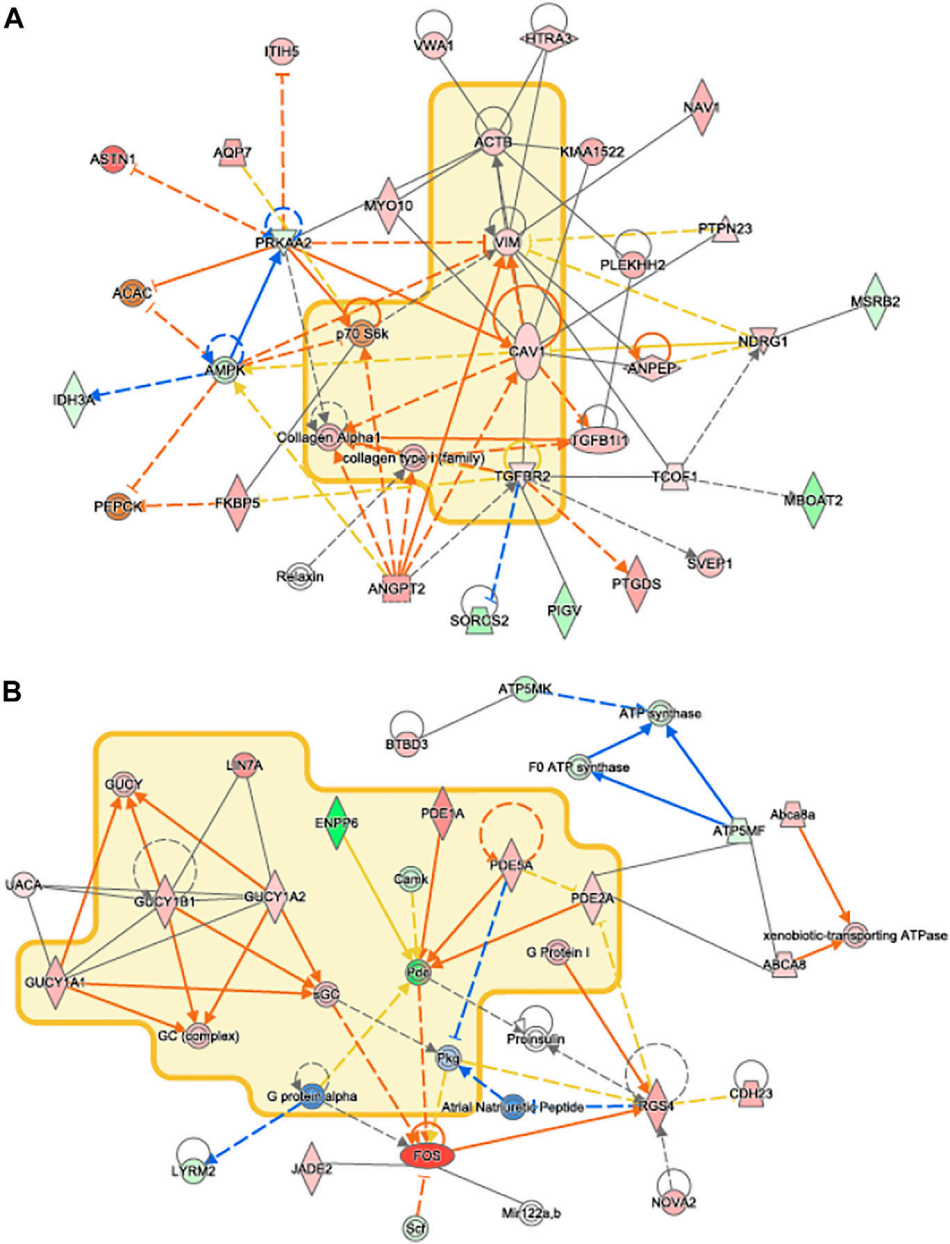
IPA Network Analysis. Novel networks detected by IPA’s network analysis. Molecules in the Idiopathic Pulmonary Fibrosis and Cardiac Hypertrophy Signaling pathways are surrounded by a yellow cloud in **(A)** and **(B)**, respectively. The genes of proteins significantly upregulated or downregulated in our data set are shaded in red and green, respectively. Activating and inhibitory interactions are shown by edges with pointed and flat arrowheads, respectively. Edges with no arrowhead indicate an interaction with no know activating or inhibitory effect. Edges that represent interactions predicted to be up or down regulated in our data set are colored in orange and blue, respectively. Yellow edges indicate that the direction of regulation of the interaction predicted by our data contradicts what was expected.

Network analysis of differentially expressed genes in IPA showed that upregulated genes, including members of the TGFβ1 family regulates genes associated with collagen synthesis and overlapped with idiopathic pulmonary fibrosis (Figure 8A). Genes of the PDE family, important for cardiac remodeling, overlapped with cardiac hypertrophy pathways (Figure 8B).

IPA results can be found in Supplementary Table S2. Full DeSeq2 results can be found in Supplementary Table S3.

#### *In vitro* CTC system studies

In previous work, we validated our CTC platform for 48 h under pressure overload and VO conditions with a 60 bpm cardiac cycle (Rogers et al., 2019). In this current set of experiments, we focused on VO vs. NL for 96 h of stimulation. As detailed above, the cardiac tissue constructs in the VO experiments experienced ∼18% strain and were not allowed to fully return to the 8 mm baseline length, whereas the NL constructs experienced ∼9% strain and were allowed to return to a fully unstretched state at the end of systole. The goal of CTC system studies was to develop an *in vitro* system to recapitulate the *in vivo* cardiac remodeling process in a VO state.

#### *In vitro* CTC system histology studies

**FIGURE 9:**
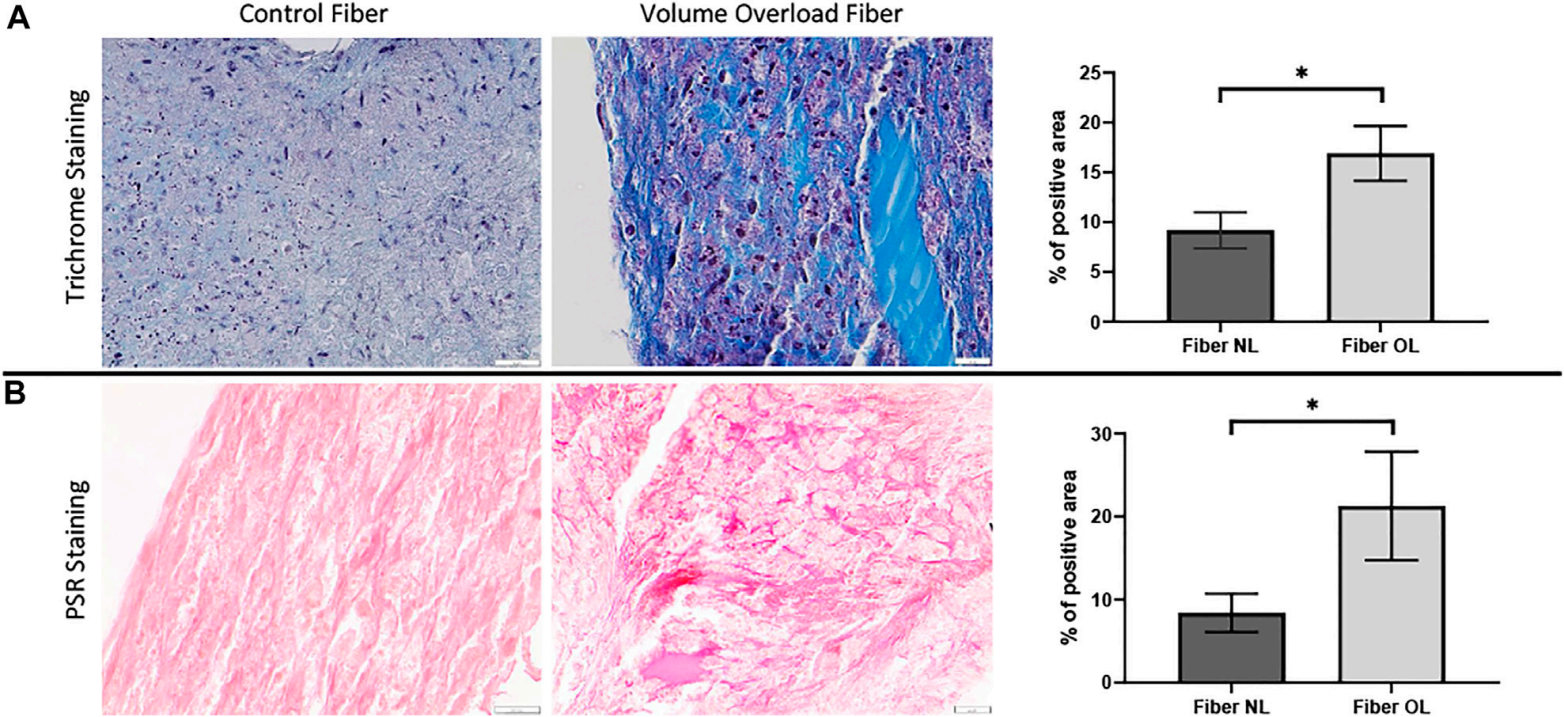
**(A)** Masson’s trichrome staining performed on volume overload fiber and control fiber (×40 magnification, scale bar 20 µm) as well as a graph showing percentage area of VO and normal fiber stained by MT staining (×40 magnification, scale bar 20 µm). **(B)** Picrosirius red staining performed on volume overload and control fibers as well as a graph showing percentage area of VO and normal fiber stained by PSR staining. (*) *p* ≤ 0.0001. N = 10 and N = 12 for the PSR and MT groups, respectively. Values represent mean ± S.D.

To analyze the morphological changes in the cardiac tissue constructs, we performed Masson’s trichrome and PSR staining. In the VO tissue constructs, we observed significantly increased collagen deposition compared to NL at 96 h (21.3% vs. 8.4%, *p* < 0.0001) (Figure 9).

#### Gene expression profiling studies

**FIGURE 10:**
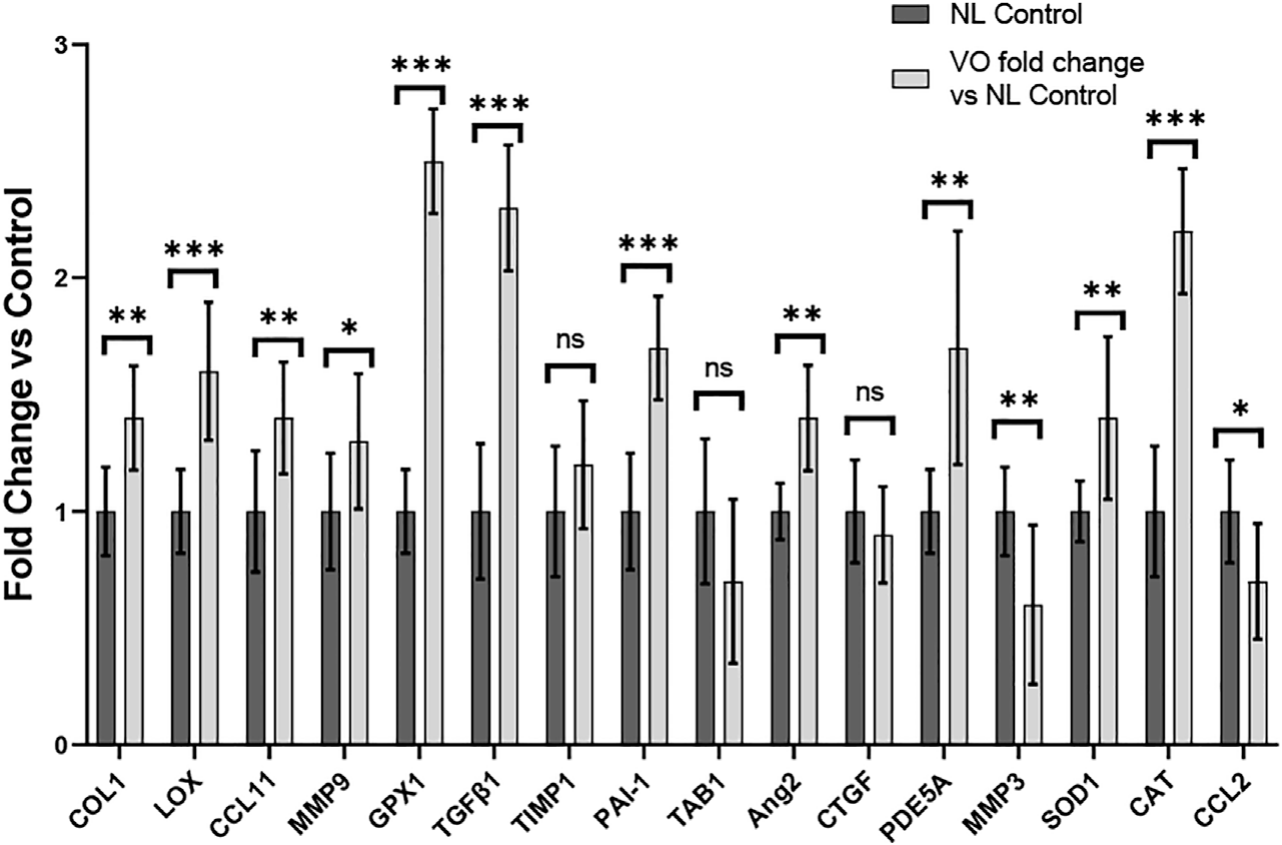
Gene expression profiles of rat myoblasts from CTC in Volume Overload versus Normal Load Control Conditions *p < 0.05, **p < 0.01, and ***p < 0.001. ns = non-significant. N = 9 and N = 5, respectively. Values represent mean ± S.D.

To evaluate the remodeling capacity of cardiac tissue constructs, we measured gene expression profiles using RT-PCR analysis, comparing samples exposed to NL to those that underwent VO conditions. We focused on genes related to inflammation and oxidative stress, extracellular matrix remodeling, and fibrosis. Genes associated with inflammation and oxidative stress (e.g., GPX1, CAT, CCL11, and SOD1), extracellular matrix remodeling (e.g., LOX), and fibrosis (e.g. COL1 and TGFβ1) were significantly upregulated in the VO vs. NL conditions (Figure 10), similar to our RT-PCR studies from our murine studies (Figure 6). Furthermore, PDE5A, a phosphodiesterase catalyzes the specific hydrolysis of cGMP to 5′-GMP and negatively impacts vasorelaxation, was also significantly elevated in the VO group. Thus, in our *in vitro* CTC system we observed significant expression of several genes that may lead to pathologic cardiac remodeling during VO stimulation. The list of primers used is listed in Supplementary Table S1. A comparison of the results between our *in vivo* and *in vitro* models can be found in Supplementary Table S4.

## Discussion

Our present study demonstrates that our mouse AVF and CTC models are promising experimental models to study the volume overload state following AVF creation and potentially complement one another. The key findings from this study are that: (1) our AVF model at 28 days demonstrated adverse cardiac remodeling with a significant increase in cardiac output and LVEDD, a decrease in EF and FS, and increased perivascular and interstitial fibrosis; (2) our CTC model with VO conditions at 96 h demonstrated increased fibrosis; (3) both our VO CTC and AVF mouse models demonstrated increased expression of genes associated with fibrosis, inflammation, and oxidative stress; and (4) transcriptomics studies from our AVF model demonstrated genes associated with pathways of fibrosis were increased in our 28 days AVF model.

Understanding the molecular mechanisms of cardiac remodeling in response to AVF creation and identifying therapeutic targets can improve cardiovascular outcomes for hemodialysis patients. Models of human cardiovascular disease, such as CTC systems, that replicate critical aspects of the onset and progression of pathological conditions can provide a viable complement to animal models and can help investigators gain a more comprehensive understanding of disease pathogenesis and aid in drug discovery and toxicity testing. Using the CTC, we have established a physiologically-relevant *in vitro* model where the VO state that occurs following AVF creation can be recapitulated and potentially used to test pharmacologic agents. Three-dimensional dynamic CTC models confer the ability to modify the hemodynamic forces experienced and generated by the left ventricle and study their effects either in isolation or combination. To recapitulate the hemodynamics of the post-AVF and sham mouse LVs using our *in vitro* CTC system, we adjusted one device fabrication parameter (membrane thickness) and two parameters of the hemodynamic loop setup (media reservoir height and resistance element tightness). As seen here, the current CTC model can exert meaningfully different, physiologically-relevant levels of strain and residual end-systolic stretch onto cardiac tissue constructs while maintaining identical intra-device pressures, allowing for faithful recapitulation of VO and NL states and eliciting similar changes in gene expression profiles and collagen deposition seen in mouse studies after just 96 h.

VO occurs in response to increased blood volume and is common in clinical conditions such as aortic regurgitation or in scenarios where excess rise in preload occurs (e.g., after AVF creation), and results in predominantly eccentric hypertrophy from thinning of the ventricular wall. While VO conditions are typically less profibrotic than pressure overload (Kong et al., 2014; Frangogiannis, 2019), particularly in the early stages of cardiac remodeling, fibrosis has not been well studied in VO. In our murine AVF model, which produces a VO state similar to that seen in humans, we demonstrated novel histological findings at 28 days post AVF creation with results exhibiting LV fibrosis in both the perivascular and interstitial regions, which recapitulates human biological findings. We next examined molecular changes in our murine AVF model with RT-PCR and confirmed that genes associated with fibrosis development such as COL1, LOX, and TGFβ1 were significantly upregulated as compared to sham-operated control mice (Figure 6). Furthermore, genes associated with oxidative stress and inflammation were also upregulated. Subsequently, we performed transcriptomics studies to identify pathways that may explain these molecular changes following AVF creation. Our results suggest that genes that inactivate mitochondrial function and activate pro-fibrotic pathways and upstream regulators may explain the physiological and histological changes seen in the LV after AVF creation (Figures 4, 5). Moreover, our IPA network analysis further suggests an important role for TGFβ1 in activation of collagen pathways and development of fibrosis and the PDE family in enhancing cardiac signaling (Figure 8).

In our CTC model, after 96 h of cardiac tissue construct stimulation, we similarly observed greater accumulation of collagen in the VO tissues as compared to the NL tissues (Figure 9). In our RT-PCR studies in our CTC samples, we found a substantial number of significantly activated profibrotic genes in the VO group vs. NL condition, such as COL1, LOX, and TGFβ1, which was similar to our murine AVF model (Figure 10). Our work using the CTC shows that the 96 h time point recapitulates many aspects seen in our AVF murine model at 28 days. The CTC can potentially serve as a model to characterize acute and chronic effects of VO on cardiac remodeling and may prove useful in evaluating therapies to mitigate adverse cardiac remodeling and complement our animal AVF model.

Our study has several limitations. First, our animal studies were performed in healthy mice and not CKD mice, so it does not fully replicate the environment seen in human AVF creation. Second, our study utilizes undifferentiated rat H9c2 cells and primary human dermal fibroblasts, rather than mature human cardiomyocytes and ventricular fibroblasts. Though these cell types do not fully replicate the function of resident cardiac cells and may differ in their responses to volume overload, we opted to utilize them for a number of reasons. Undifferentiated H9c2s have been used in numerous *in vitro* models of cardiac fibrosis and hypertrophy (Guan et al., 2017; Xu et al., 2021; Abdulrahman et al., 2022; Anwaier et al., 2022), and while it has been demonstrated that H9c2s differentiated further toward a mature cardiomyocyte phenotype demonstrate changes in metabolism and expression of cardiomyocyte-specific proteins (Pereira et al., 2011), we hoped to maintain comparability to our past work and models such as those cited above that utilized the cells in their undifferentiated state. Though normal human dermal fibroblasts have not enjoyed widespread use in modeling cardiac fibrosis, they are frequently employed as the stromal cell component in the manufacture of engineered heart tissues (Ronaldson-Bouchard et al., 2019; Tsuruyama et al., 2019; Kikuchi et al., 2022). Both cell types are also easier and less resource-intensive to maintain in culture than human cardiomyocytes and ventricular fibroblasts, which allowed us greater freedom to optimize other aspects of the model. In future studies, we will evaluate human induced pluripotent stem cell derived cardiomyocytes (hiPSC-CMs) cultured within the CTC with primary ventricular cardiac fibroblasts to more faithfully model human cardiac tissue remodeling in response to altered hemodynamics. Third, we did not evaluate what effect anti-fibrotic therapeutics, such as AT1 receptor blockers, would have on preventing or reversing the pro-fibrotic state exhibited by the VO tissue constructs in our CTC model. Fourth, our molecular analysis is based on PCR and RNAseq analysis and does not include protein expression data. Finally, both our murine AVF studies and CTC studies included only one time point, so we were not able to access the longitudinal changes that occur following volume overload conditions. In future studies, we plan to study additional time points.

## Conclusion

Our murine AVF model recapitulates similar features of cardiac remodeling following human AVF creation with development of cardiac fibrosis at 28 days and activation of profibrotic molecular pathways. Furthermore, our CTC model also recapitulates many histologic and molecular changes exhibited by our murine AVF model in VO conditions. Thus, our two models could complement one another and prove useful in furthering our understanding of the characteristics of hemodynamic stress associated with cardiac remodeling in a volume overload state.

## Data Availability

The data presented in the study are deposited in the SRA repository, accession number PRJNA892911 https://www.ncbi.nlm.nih.gov/bioproject/PRJNA892911/.

## Glossary

AVF: arteriovenous fistula
BCTM: biomimetic cardiac tissue model
BPM: beats per minute
BSA: bovine serum albumin
CO: cardiac output
CTC: cardiac tissue chip
DMEM: Dulbecco’s modified Eagle’s medium
ECM: extracellular matrix
EDV: end diastolic volume
ESV: end systolic volume
EF: ejection fraction
ESKD: end stage kidney disease
FBS: fetal bovine serum
FDR: false discovery rate
FS: fractional shortening
HR: heart rate
IPA: ingenuity pathway analysis
IVS;d: interventricular septal end-diastole
LV: left ventricle
LVEDD: left ventricular end-diastolic diameter
LVID; d: left ventricular end-diastolic inner dimension
LVID; s: left ventricular end-systolic inner dimension
LVPW, d: left ventricular posterior wall end-diastole
MT: Masson’s Trichrome
NL: normal load
PDMS: polydimethylsiloxane
PSR: picrosirius red
RT-PCR: real time polymerase chain reaction
VO: volume overload
