# Supplementary figures and images for: Biomimetic cardiac tissue chip and murine arteriovenous fistula models for recapitulating clinically relevant cardiac remodeling under volume overload conditions
Source: Front Bioeng Biotechnol. 2023 Feb 16;11:1101622. doi: 10.3389/fbioe.2023.1101622 (PMC9978753; doi:10.3389/fbioe.2023.1101622)

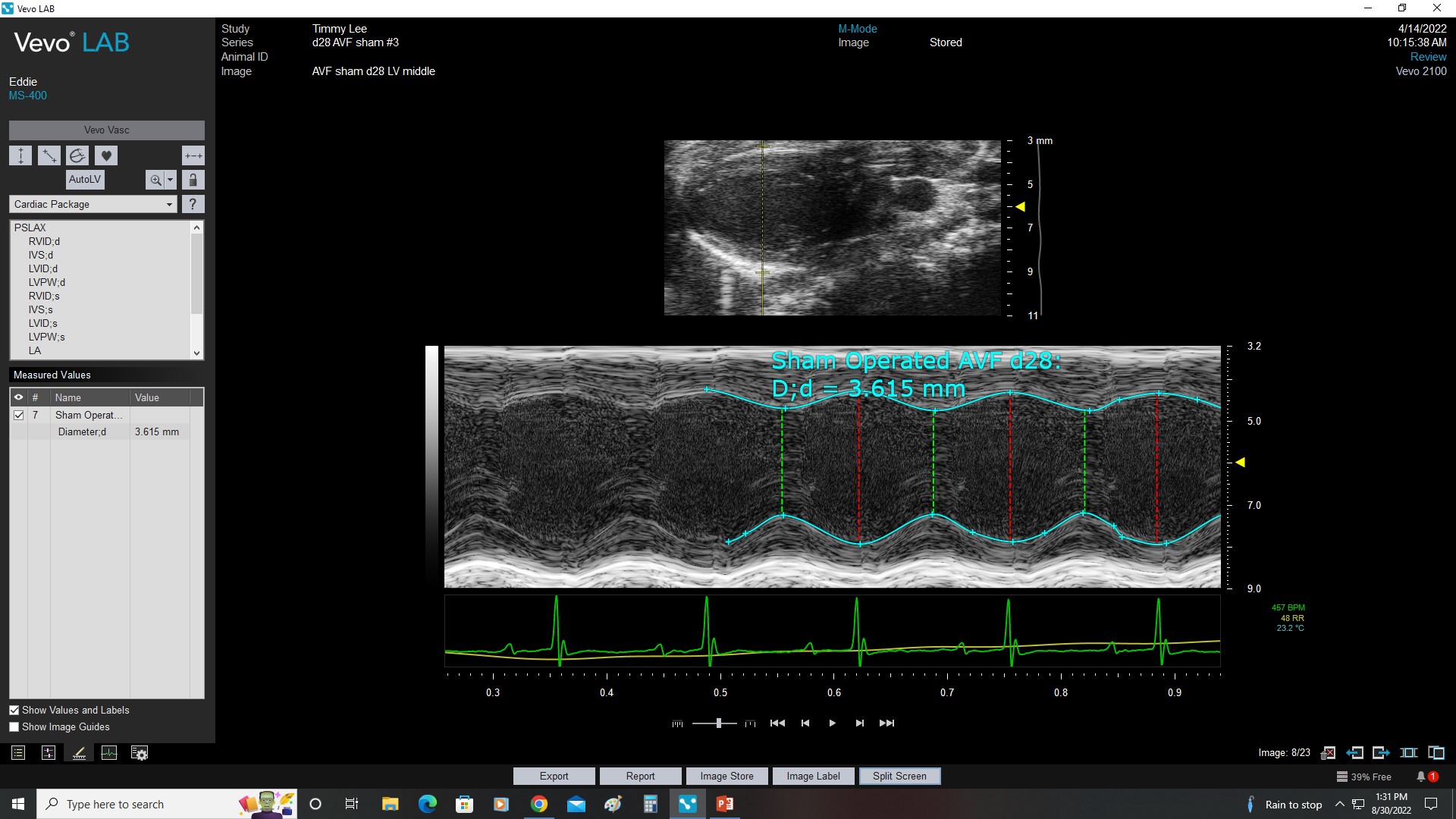

Supplement: Supplementary file 2 [file Image3.JPEG]

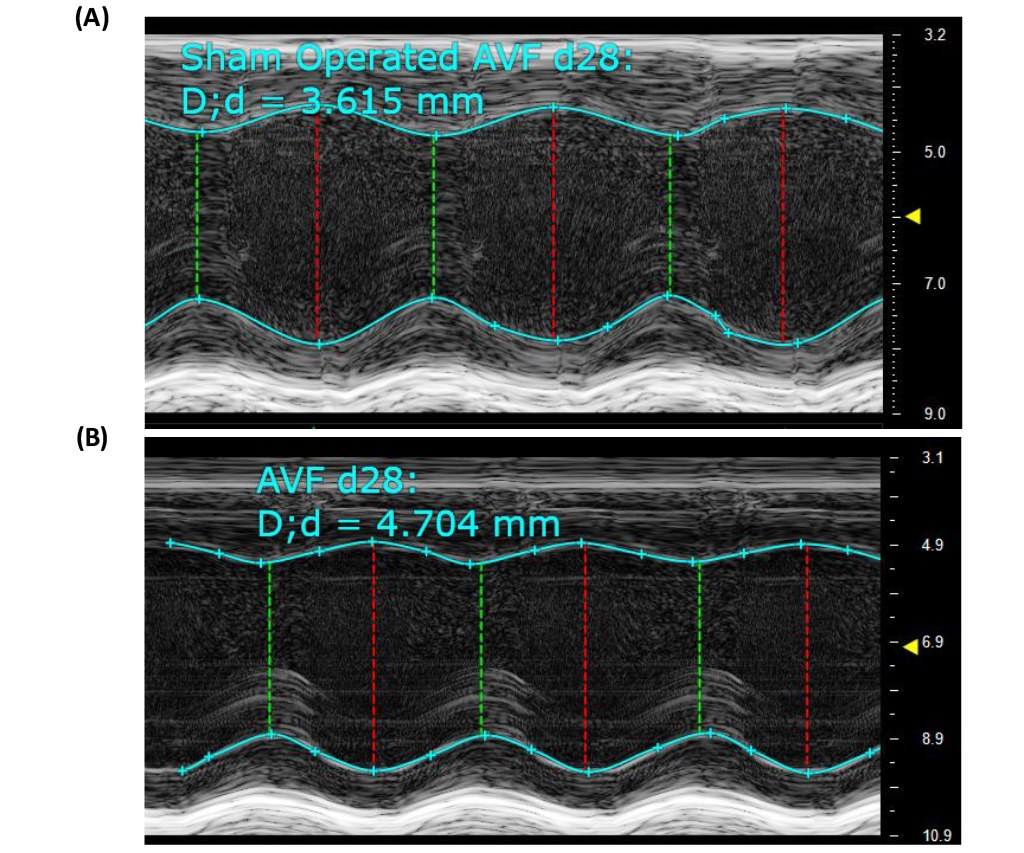

Supplement: Supplementary file 3 [file Image1.TIFF]

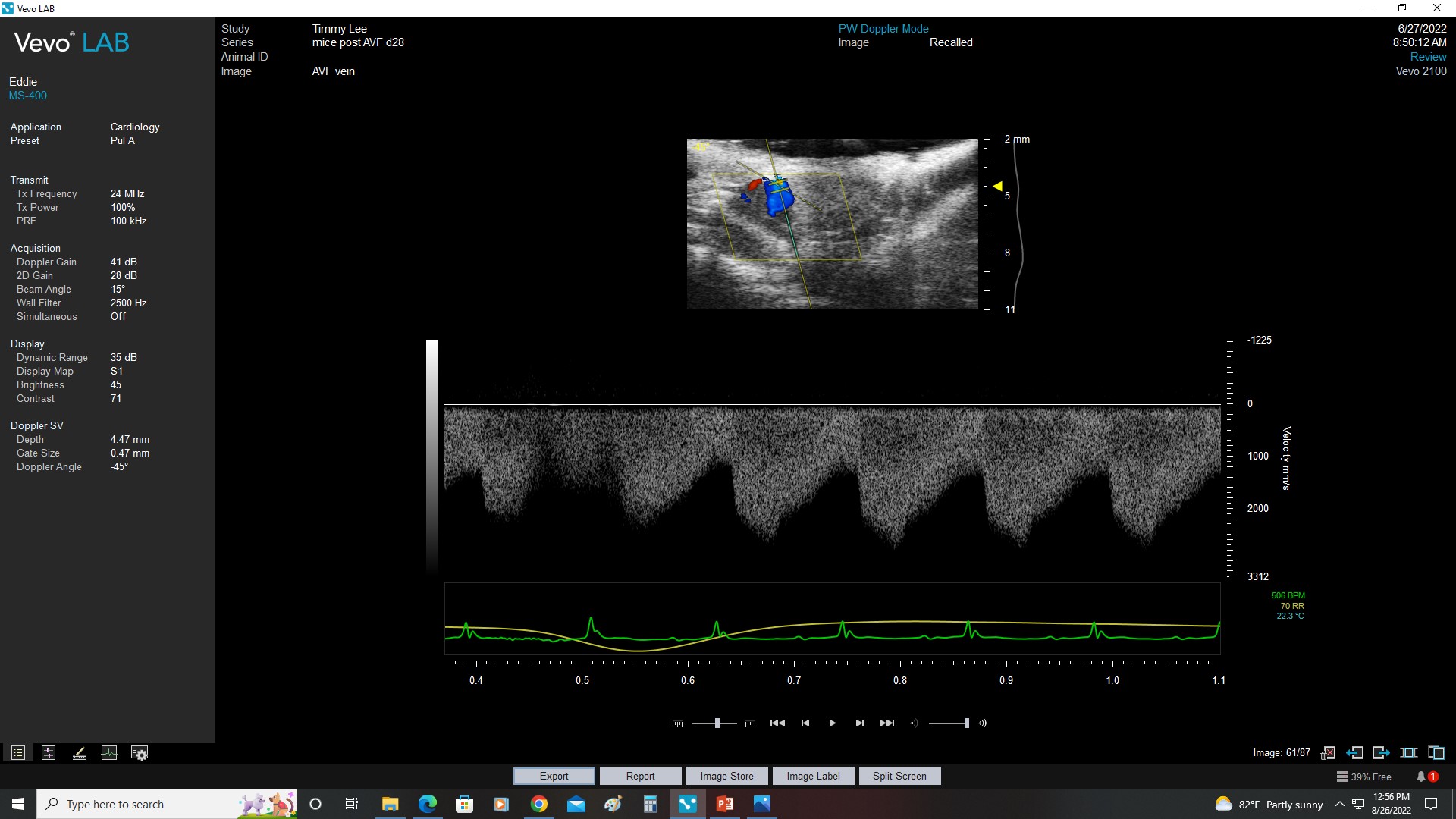

Supplement: Supplementary file 6 [file Image9.JPEG]

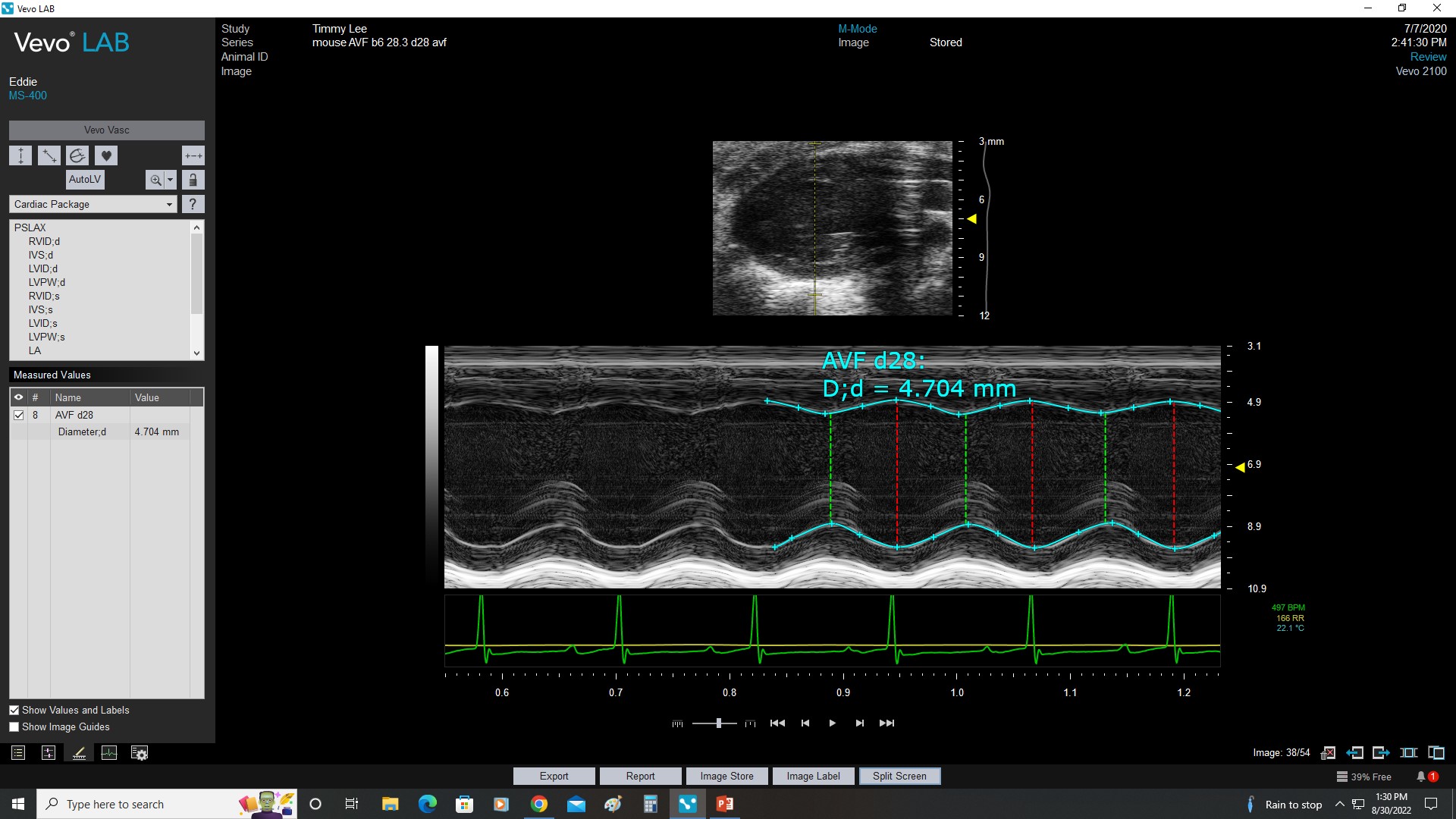

Supplement: Supplementary file 7 [file Image4.JPEG]

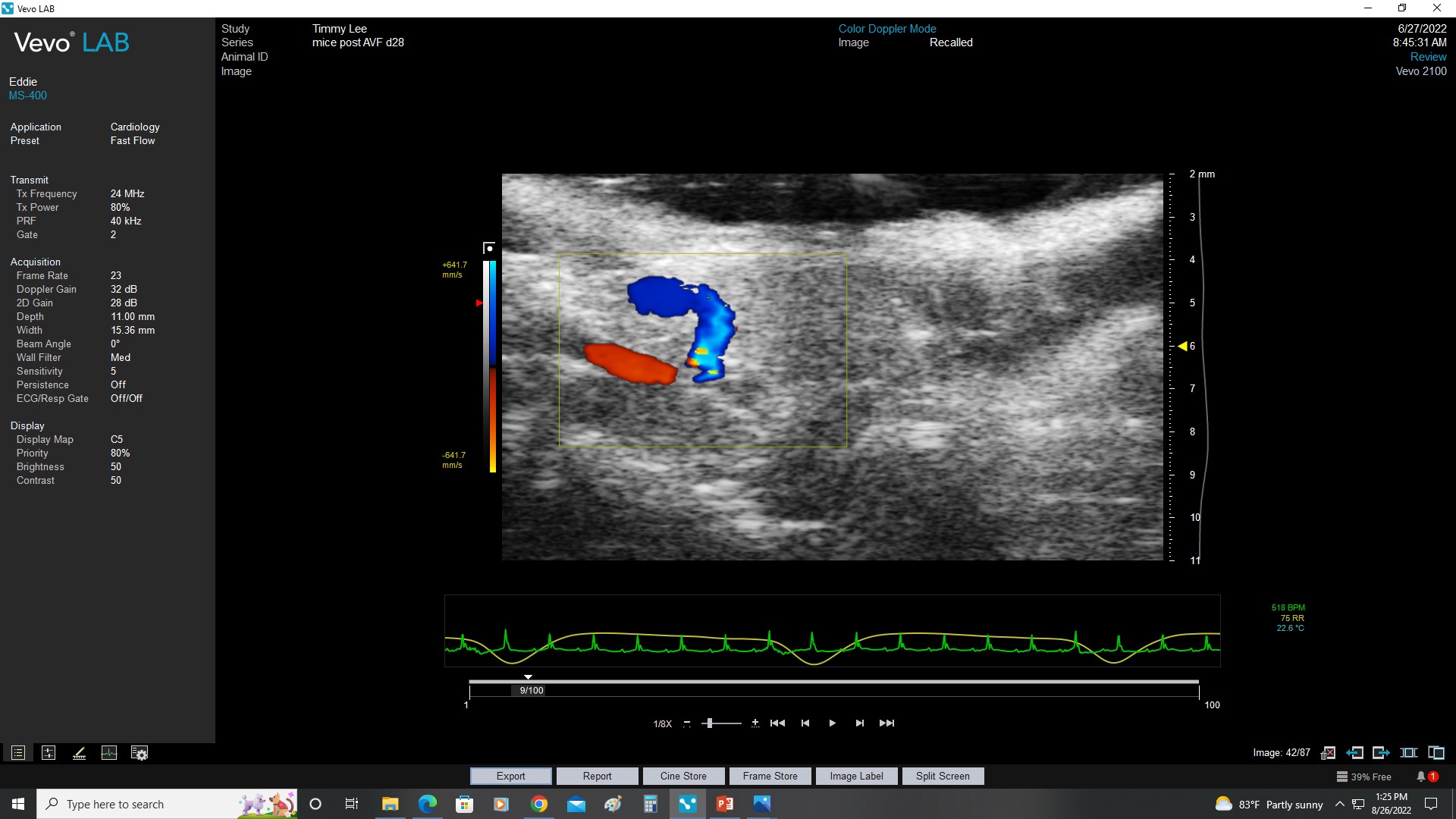

Supplement: Supplementary file 8 [file Image7.JPEG]

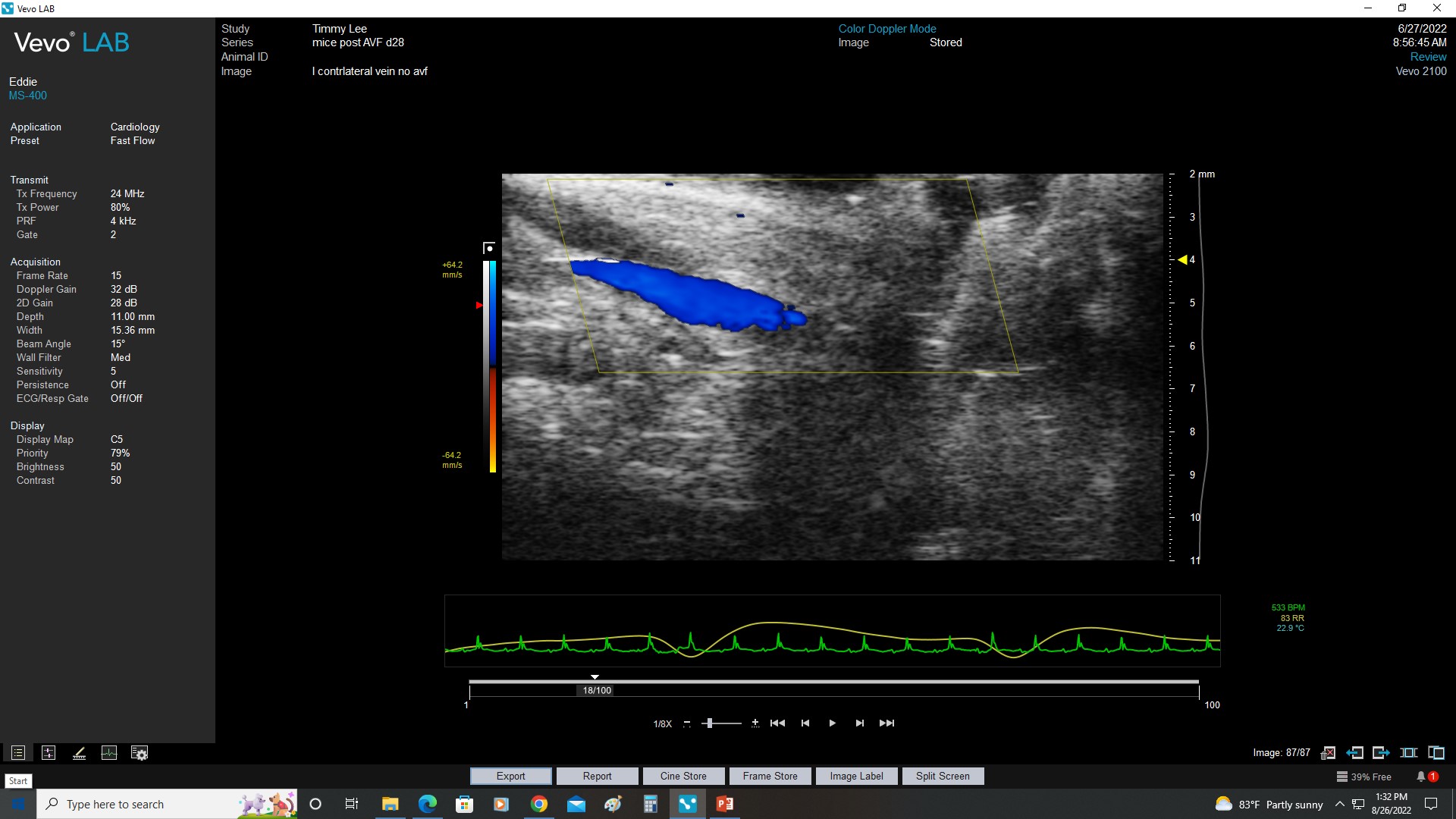

Supplement: Supplementary file 9 [file Image5.JPEG]

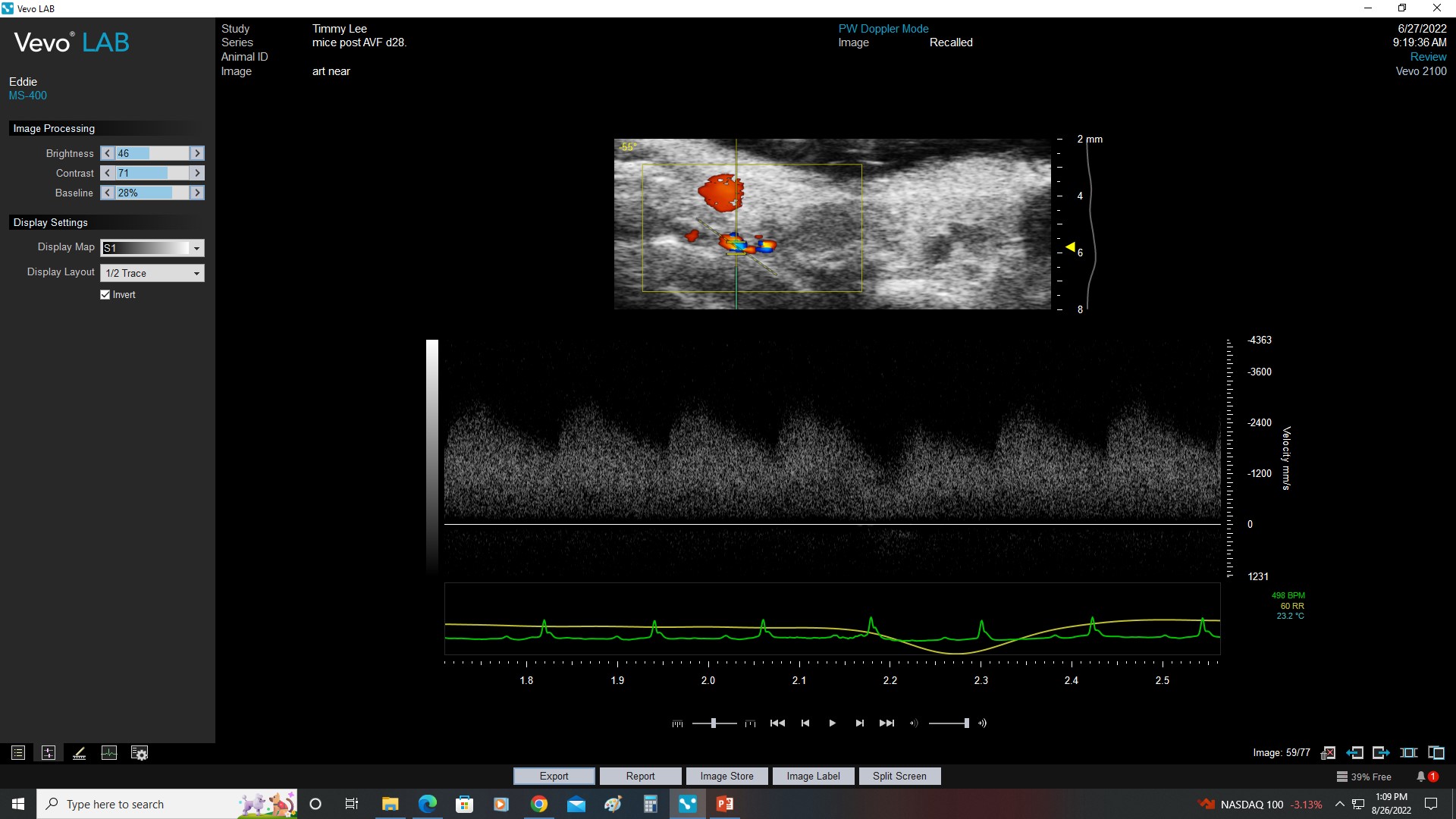

Supplement: Supplementary file 10 [file Image10.JPEG]

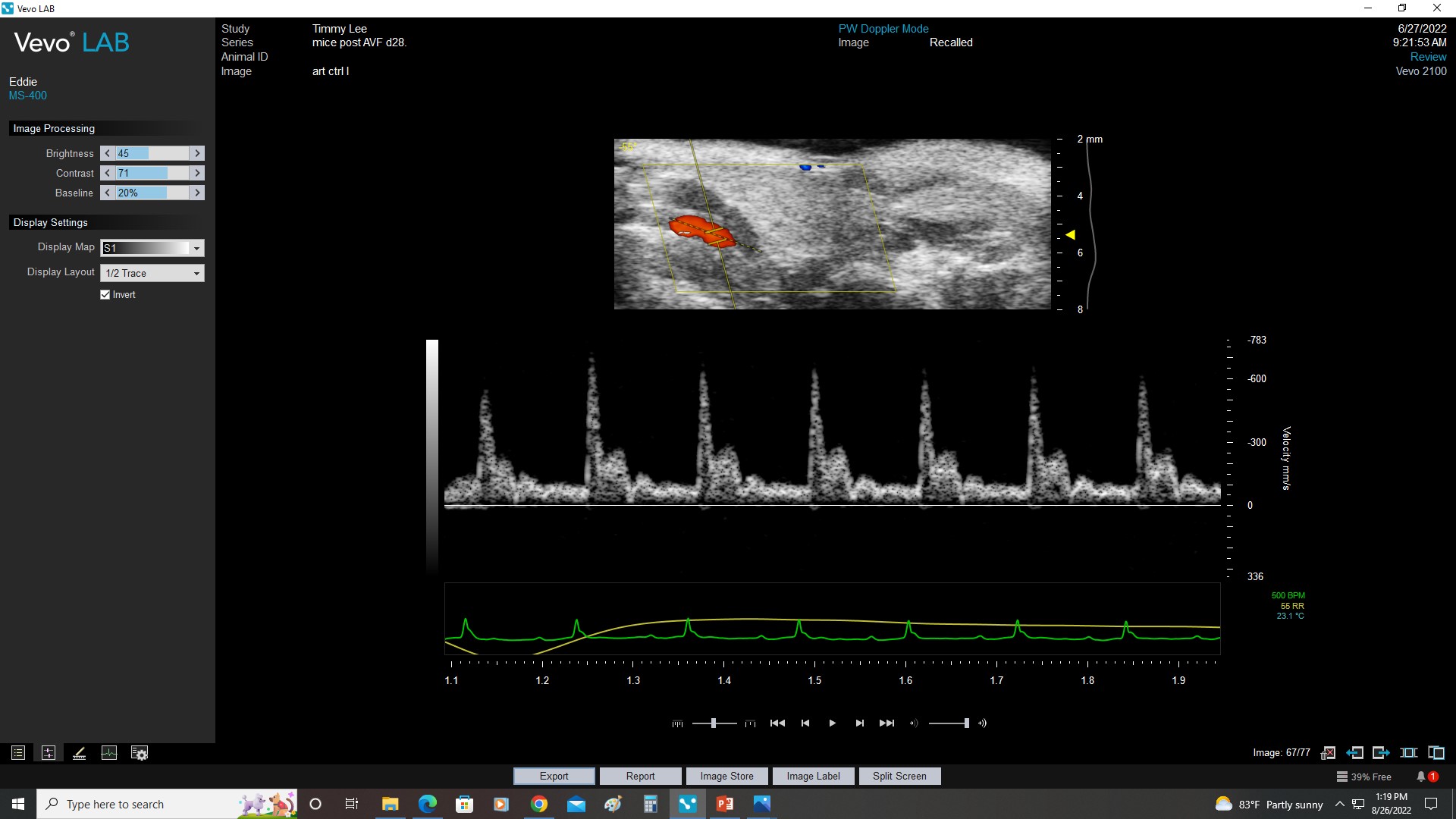

Supplement: Supplementary file 11 [file Image11.JPEG]

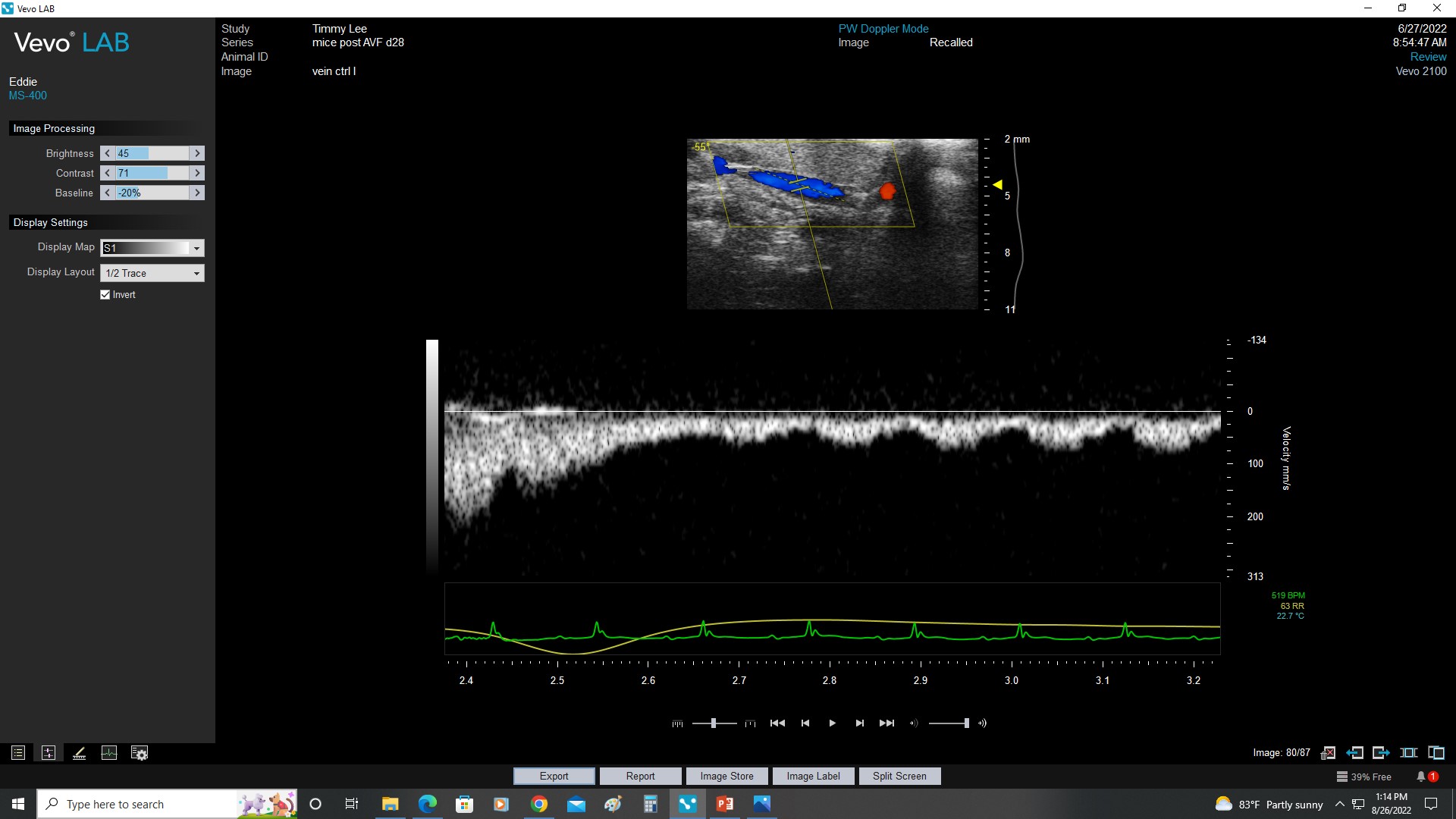

Supplement: Supplementary file 13 [file Image8.JPEG]

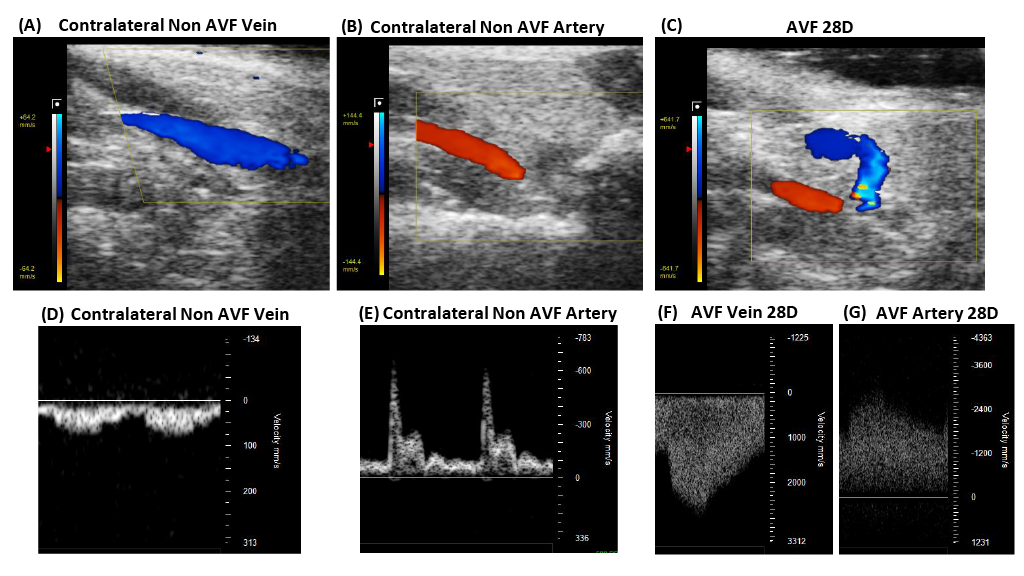

Supplement: Supplementary file 14 [file Image2.TIFF]

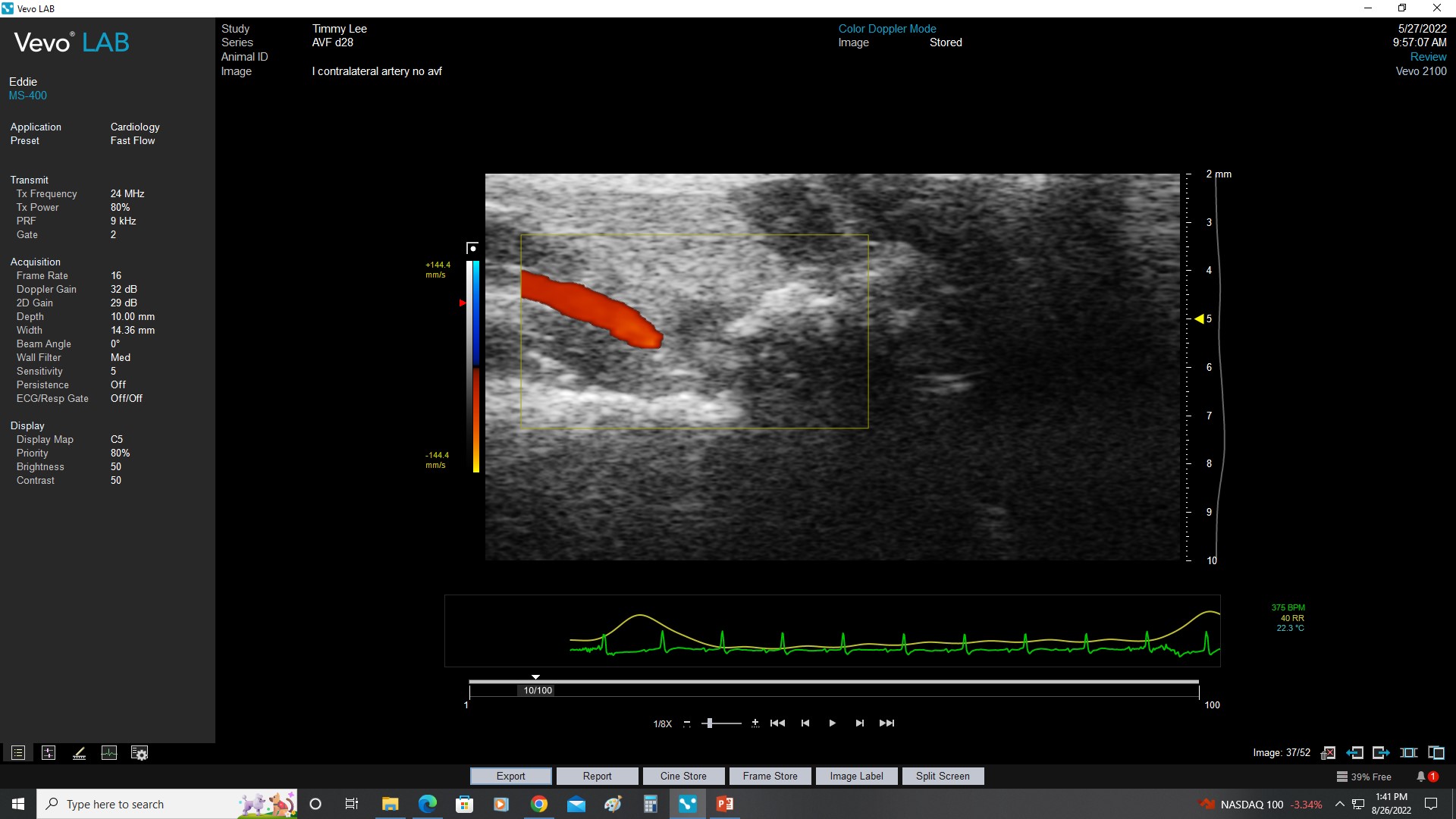

Supplement: Supplementary file 15 [file Image6.JPEG]
