# Supplementary material for: Biomimetic cardiac tissue chip and murine arteriovenous fistula models for recapitulating clinically relevant cardiac remodeling under volume overload conditions
Source: Front Bioeng Biotechnol. 2023 Feb 16;11:1101622. doi: 10.3389/fbioe.2023.1101622 (PMC9978753; doi:10.3389/fbioe.2023.1101622)
Supplement: Supplementary file 5 [file Table1.DOCX]

**Supplementary Table 1:**

|  | Rat primers | RefSeq | Mouse primers | RefSeq |
| --- | --- | --- | --- | --- |
| AGT | Rn00593114_m1 | NM_134432.2 | Mm00599662_m1 | NM_007428.3 |
| ANG2 | Rn02349499_g1 | NM_001012359.1 | Mm006957574_s1 | NM_007449.2 |
| ATG3 | Rn00471287_m1 | NM_134394.2 | Mm00471287_m1 | NM_026402.3 |
| CAT | Rn01512560_m1 | NM_012520.2 | Mm00437992_m1 | NM_009804.2 |
| CCL2 | Rn00580555_m1 | NM_031430.1 | Mm00441242_m1 | NM_011333.3 |
| CCL11 | Rn00569995_m1 | NM_019205.1 | Mm00441238_m1 | NM_011330.3 |
| COL1A1 | Rn01463848_m1 | NM_053304.1 | Mm00801666_g1 | NM_007742.3 |
| CTGF | Rn01537277_g1 | NM_022266.2 | Mm01192933_g1 | NM_010217.2 |
| FN1 | Rn00569575_m1 | NM_019143.2 | Mm01256744_m1 | NM_001276408.1 |
| GAPDH | Rn01749022_g1 | NM_017008.4 | Mm99999915_g1 | NM_001289726.1 |
| GPX1 | Rn00577994_g1 | NM_030826.3 | Mm04207457_g1 | NM_008160.6 |
| LOX | Rn01491829_m1 | NM_017061.2 | Mm00495386_m1 | NM_001286181.1 |
| MMP3 | Rn00591740_m1 | X02601.1 | Mm00440295_m1 | NM_010809.1 |
| MMP9 | Rn00579162_m1 | NM_031055.1 | Mm00442991_m1 | NM_013599.3 |
| PAI-1 | Rn01481341_m1 | NM_012620.1 | Mm00435858_m1 | NM_008871.2 |
| PDGFb | Rn01502596_m1 | NM_031524.1 | Mm00440677_m1 | NM_011057.3 |
| PDE2A | Rn00676004_g1 | NM_001143847.1 | Mm01136644_m1 | NM_001008548.4 |
| PDE5A | Rn01639345_m1 | NM_133584.1 | Mm00463177_m1 | NM_153422.2 |
| SMAD3 | Rn00565331_m1 | NM_013095.3 | Mm01170760_m1 | NM_016769.4 |
| SOD1 | Rn00566938_m1 | NM_017050.1 | Mm01344233_g1 | NM_011434.1 |
| TAB1 | Rn01504766_m1 | NM_001109976.2 | Mm01298616_m1 | NM_025609.2 |
| TGF beta | Rn00572010_m1 | NM_02157.8 | Mm01178820_m1 | NM_011577.1 |
| TIMP1 | Rn01430874_g1 | NM_053819.1 | Mm00441818_m1 | NM_001044384.1 |
