# Supplementary material for: Biomimetic cardiac tissue chip and murine arteriovenous fistula models for recapitulating clinically relevant cardiac remodeling under volume overload conditions
Source: Front Bioeng Biotechnol. 2023 Feb 16;11:1101622. doi: 10.3389/fbioe.2023.1101622 (PMC9978753; doi:10.3389/fbioe.2023.1101622)
Supplement: Supplementary file 12 [file Table4.DOCX]

**Supplementary Table 4**

| **Gene** | **Animal Model** | **CTC** | **Similar Change in Gene Expression Profile? (y/n)** |
| --- | --- | --- | --- |
| COL1 | Significant Upregulation | Significant Upregulation | Y |
| LOX | Significant Upregulation | Significant Upregulation | Y |
| CCL11 | Significant Upregulation | Significant Upregulation | Y |
| MMP9 | Significant Upregulation | Significant Upregulation | Y |
| TGFβ1 | Significant Upregulation | Significant Upregulation | Y |
| TIMP1 | Significant Upregulation | Non-significant Upregulation | N |
| PAI-1 | Significant Upregulation | Significant Upregulation | Y |
| TAB1 | Significant Upregulation | Non-significant Downregulation | N |
| Ang2 | Significant Upregulation | Significant Upregulation | Y |
| CTGF | Significant Upregulation | Non-significant Downregulation | N |
| PDE5A | Significant Upregulation | Significant Upregulation | Y |
| MMP3 or 2? | Significant Upregulation | Significant Downregulation | N* |
| SOD1 | Significant Upregulation | Significant Upregulation | Y |
| CAT | Significant Upregulation | Significant Upregulation | Y |
| CCL2 | Significant Upregulation | Significant Downregulation | N* |

* The expression profile for one model was significant and opposite of the other model (e.g. significantly upregulated in one model and significantly downregulated in the other).
